# Development of pathogenicity predictors specific for variants that do not comply with clinical guidelines for the use of computational evidence

**DOI:** 10.1186/s12864-017-3914-0

**Published:** 2017-08-11

**Authors:** Elena Álvarez de la Campa, Natàlia Padilla, Xavier de la Cruz

**Affiliations:** 1 Research Unit in Translational Bioinformatics, Vall d’Hebron Institute of Research (VHIR), Universitat Autònoma de Barcelona, Barcelona, Spain; 2 Department of Molecular Genomics, Instituto de Biología Molecular de Barcelona (IBMB), Consejo Superior de Investigaciones Científicas (CSIC), Barcelona, Spain; 3 ICREA, Barcelona, Spain

**Keywords:** In silico pathogenicity predictors, Protein sequence variants, Molecular diagnostics, Missense variants, Next-generation sequencing

## Abstract

**Background:**

Strict guidelines delimit the use of computational information in the clinical setting, due to the still moderate accuracy of in silico tools. These guidelines indicate that several tools should always be used and that full coincidence between them is required if we want to consider their results as supporting evidence in medical decision processes. Application of this simple rule certainly decreases the error rate of in silico pathogenicity assignments. However, when predictors disagree this rule results in the rejection of potentially valuable information for a number of variants. In this work, we focus on these variants of the protein sequence and develop specific predictors to help improve the success rate of their annotation.

**Results:**

We have used a set of 59,442 protein sequence variants (15,723 pathological and 43,719 neutral) from 228 proteins to identify those cases for which pathogenicity predictors disagree. We have repeated this process for all the possible combinations of five known methods (SIFT, PolyPhen-2, PON-P2, CADD and MutationTaster2). For each resulting subset we have trained a specific pathogenicity predictor. We find that these specific predictors are able to discriminate between neutral and pathogenic variants, with a success rate different from random. They tend to outperform the constitutive methods but this trend decreases as the performance of the constitutive predictor improves (e.g. with PON-P2 and PolyPhen-2). We also find that specific methods outperform standard consensus methods (Condel and CAROL).

**Conclusion:**

Focusing development efforts on the case of variants for which known methods disagree we may obtain pathogenicity predictors with improved performances. Although we have not yet reached the success rate that allows the use of this computational evidence in a clinical setting, the simplicity of the approach indicates that more advanced methods may reach this goal in a close future.

**Electronic supplementary material:**

The online version of this article (doi:10.1186/s12864-017-3914-0) contains supplementary material, which is available to authorized users.

## Background

The application of NGS in the clinical setting is limited, among other things, by our inability to accurately pinpoint the causative variant of a patient’s condition from the set of variants identified in sequencing experiments [1]. Frequently, this is due to a lack of information on the pathogenicity of these variants. In this situation, pathogenicity predictors, designed to estimate the damage caused by sequence variants [2, 3], can provide valuable information. For variants resulting in amino acid substitutions, these tools combine properties that measure different aspects of protein structure/function. For example, some of the properties (like hydrophobicity or volume differences) are related to changes in protein stability upon mutation, while others indicate whether the functional site of the protein has been damaged [2]. Using this information, in silico predictors produce a numerical score that is transformed into a binary prediction (pathogenic/neutral) through the use of a decision threshold. The accuracy of these predictions is around 80% [2, 3]. Although this value is not a fundamental threshold limiting the usage of in silico tools in the clinical, this kind of application was not initially advocated [3–5]. However, this situation is changing due to three facts. First, the drop in sequencing costs is leaving variant interpretation as one of the main bottlenecks in clinical applications of NGS [1] thus creating an important pressure for finding strategies that alleviate this problem. Second, and further in this direction, clinical users increasingly consider the possibility of using pathogenicity predictions as supporting evidence that can be combined with medical data to support diagnostic decisions [6–8]. This view has been facilitated by the clarification of the probabilistic nature of computational evidence [9]. And, third, the fact that the success rate of pathogenicity predictors remains around 80%, regardless of the technical differences between them [2, 3], indicates that these tools recognize a signal common to many pathogenic variants but absent in neutral ones [2, 3].

In this scenario, where pathogenicity predictions can be useful but are still imperfect, the idea of scoring variants with several predictors is gaining support in healthcare applications [3, 9, 10]. The underlying rationale is that because different methods implement (partially) complementary representations of the variant’s impact, coincidence in their predictions would be reinforcing. This idea is included in the guidelines for variant interpretation of the American College of Medical Genetics and Genomics (ACMG) and the Association for Molecular Pathology (AMP) [11]. There, the application of more than one predictor is considered advantageous and, to combine the resulting evidence, it is proposed that "*If all of the in silico programs tested agree on the prediction, then this evidence can be counted as supporting. If in silico predictions disagree, however, then this evidence should not be used in classifying a variant*". The value of this type rule (to which we will refer to as the coincidence rule) has been observed in different works [12–14]. However, when pathogenicity predictions disagree this rule will result in the rejection of computational evidence and, consequently, in a reduction of the data available to make medical decisions. Not only this, this effect will affect an increasing number of variants if we combine more predictors in a quest for higher reliability. In this work, we address this problem and study whether we can develop specific, competitive pathogenicity predictors for those variants for which known methods give contradictory results. To this end, we have developed a series of neural network-based predictors using a dataset of pathogenic and neutral variants for which five known predictors (SIFT, PolyPhen-2, CADD, PON-P2 and MutationTaster2) disagreed in their results (Additional file 1: Figure S1; this set will be called PRDIS). To build our tools we have explored different options (Additional file 2: Figure S2), including the use of two neural networks (NN) -a model with no hidden layer and one with a single hidden layer and two nodes, and different combinations of input attributes (using prediction scores and molecular/evolutionary properties). Note: since in this work we will frequently compare different sets of predictors, to avoid confusion we will refer to SIFT, PolyPhen-2, CADD, PON-P2 and MutationTaster2 as reference tools/predictors/methods, and to Condel and CAROL as consensus tools/predictors/methods.

The results obtained show that there is a high number of variants, between 10% and 45% of the cases studied, for which contradictory predictions are obtained. For these variants we find that we can build specific pathogenicity predictors with non-random success rates. In fact, the performance of these specifically trained tools generally improves on that of the reference tools used (SIFT, PolyPhen-2, CADD, PON-P2 and MutationTaster2) and on that of consensus pathogenicity predictors (Condel and CAROL). Finally, we provide a global view of what prediction performance can be reached when combining in a hybrid method the coincidence (or ACMG/AMP) rule and the predictors for PRDIS.

## Methods

Note. We will use the terms ‘specific’ or ‘PRDIS specific’ predictors for those predictors obtained using variants from PRDIS only.

### General protocol for building the PRDIS specific predictors

The goal of this work is to study whether we can obtain better pathogenicity predictions by developing methods specific for subsets of the variant. More precisely, in this work we have used the coincidence rule to partition our set of variants (Additional file 1: Figure S1) and develop specific predictors for PRDIS (Additional file 2: Figure S2), the subset of variants for which the reference predictors disagree. We have studied this problem for all possible combinations of five reference predictors (Additional file 2: Figure S2): SIFT, PolyPhen-2, PON-P2, CADD and MutationTaster2. For each of these combinations, we will obtain a PRDIS and this set will be used for training a neural network predictor following a standard protocol that has been described in our previous work [2, 15, 16]. For each PRDIS, this protocol is divided into three steps: (i) characterization of variants with several properties, (ii) build a neural network model for variant prediction and (iii) estimate its performance. Below we describe these steps, although more information can be found in our previous work [2, 15, 16].

### Variant datasets

The development of the pathogenicity predictors PRDIS required, in a first step, to build an initial set of pathological and neutral variants; in a second step, this set of variants is processed to give the PRDIS sets that will be used to derive the predictors tested in this work. Below, we devote a specific section to each of these two steps.

### The initial variant dataset

This dataset, constituted by pathological and neutral variants, was obtained following commonly used procedures [2, 15, 16]. Pathological variants were retrieved from UniProt [17] and corresponded to sequence variants labeled as “Disease” in Humsavar (version 06-JUL-2016). However, not all of them were included in our initial dataset; we removed those variants from proteins contributing less than 30 independent variants to Humsavar. For example, if for a protein there were only two known variants in Humsavar, none of them was included in our initial dataset. On the contrary, if for a protein there were 31 variants listed in Humsavar, all of them were included in our initial dataset. The reason for this filter is to avoid the large imbalances between the number of pathological and neutral variants in the dataset, caused by proteins contributing few pathological but many neutral variants. The threshold (thres) value of 30 pathological variants per protein was chosen after exploring the dependence of the ratio of neutral to pathological variants on different thres values: 12.5 (thres = 0), 6.3 (thres = 5), 4.8 (thres = 10), 2.8 (thres = 30) and 2.0 (thres = 50). On the basis of our previous work (Fig. 4 in [15]), where we found that for ratios above 5 the sample imbalance becomes increasingly difficult to correct, we chose a conservative threshold (thres = 30) for this work. Higher values were discarded because the number of proteins dropped substantially, e.g. for thres = 50, only 130 proteins contributed variants to the dataset, compared to 228 for thres = 30. At the end of the process, we obtained 15,723 pathogenic variants, distributed over a total of 228 proteins.

For neutral variants, we used the homology-based model described in our previous work [2, 15, 16], where variants are obtained from a multiple sequence alignment (MSA) for each protein family. More precisely, they correspond to those sequence deviations from the human representative observed in close homologs (sequences from other species > = 95% identical to the human one) [18]. The technical steps are well described in Riera et al. [15]. Here, we briefly summarize them. First, for each of the 228 proteins we retrieved their sequence from UniProt and used it to query UniRef100 (06-JUL-2016) [19], running a PsiBlast [20] query (e-value 0.001, two iterations). From this output, we eliminated those sequences less than 40% identical to the human protein. Second, the remaining sequences were aligned with Muscle [21]. And third, we collected all the deviations from the human sequence found in homologs > = 95% sequence identity. These deviations constituted the set of neutral variants for this protein. Following this protocol for the 228 proteins, we obtained a total of 43,651 neutral variants. Together with the patological cases, we obtained a set of 59,442 variants spread over 228 proteins, that we called VS228.

An annotated list of the variants in VS228, plus the pathogenicity predictions for the tools used in this work are provided as Additional file 3 (pathological variants) and Additional file 4 (neutral variants).

To check the reach of the conclusions in this work for proteins not represented in VS228, we employed those variants discarded when building VS228 because their proteins did not have 30 or more cases. The new dataset, which was not utilized during the training of our predictors, was constituted by a total of 322,270 variants (29,259 pathological and 293,011 neutral) spread over 2168 proteins. This independent, validation dataset was called VS2168. Note that in this set pathogenic variants, apart from UniProt [17], were also obtained from HGMD Professional [22], to which we have recently bought a subscription.

### The PRDIS variant datasets

As explained at the start of the Materials and Methods, we tried different versions of the coincidence rule, each corresponding to one of the combinations of five reference methods (SIFT, PolyPhen-2, PON-P2, CADD and MutationTaster2). Application of this rule to VS228 (Additional file 1: Figure S1) was used to produce a given PRDIS. Repeated application of all possible versions of the rule results in all the PRDIS used in this work (Additional file 2: Figure S2).

Equivalent PRDIS datasets were also obtained from VS2168. They were used to test if the conclusions reached with VS228 also hold for proteins (and their variants) not included in the development of our predictors.

### Characterization of variants in terms of discriminant features

We tried two different sets of features to characterize variants for building our specific methods (Additional file 2: Figure S2). In one we only used the scores of the reference predictors employed to include the variant in the PRDIS (Additional files 1, 2: Figures S1, S2). For example, when the PRDIS was built using SIFT and PON-P2, we used the SIFT and PON-P2 scores as input for our predictor; when the PRDIS was built using PolyPhen-2, CADD and PON-P2, then our input was constituted by the scores of these three methods; etc. In the second set, we enriched the previous scores with three additional properties: the element of the Blosum62 matrix [23] corresponding to the amino acid replacement and two properties related to the sequence conservation pattern at the variant locus. The first was Shannon’s entropy; it is equal to -Σ_i_p_i_.log(p_i_), where the index i runs over all the amino acids at the variant’s MSA column. The second property was the value of the position-specific scoring matrix [15, 24] for the native amino acid, pssm_nat_, which is equal to log(f_nat,i_/f_nat,MSA_), where f_nat,i_ and f_nat,MSA_ are the frequencies of the native amino acid at the variant locus i and in the whole alignment, respectively. Both Shannon’s entropy and the position-specific scoring matrix element were computed from the MSA of the protein family.

### Building the specific predictors

All our predictors were built with WEKA (v3.6.8) [25]. We tried two neural network models. One was the simplest neural network possible: a single-layer perceptron (WEKA defaults: L = 0.3, M = 0.2, *N* = 500, V = 0, S = 0, E = 20), with no hidden layers [26]. This model was chosen because we have used it with good results in our previous work [15, 16]. The second model was a slightly more complex neural network with one hidden layer having two nodes (WEKA parameters: L = 0.3, M = 0.2, *N* = 500, V = 0, S = 0, E = 20, H = 2). We used SMOTE [27] to correct for the imbalance between pathological and neutral variants in the training sets (not in the test/validation sets).

For each PRDIS, the whole procedure described in this section was applied to the two possible sets of features here described.

### Performance assessment

Performance estimates are obtained following a standard 5-fold cross-validation procedure, such as that described in our previous work. The success rate of the predictors was measured using six parameters [15, 16, 28, 29]: sensitivity, specificity, accuracy, positive predictive and negative predictive values, and Matthew’s correlation coefficient (MCC). They are computed as shown below.

.- Sensitivity:

$$ \frac{TP}{TP+FN} $$

.- Specificity:

$$ \frac{TN}{TN+FP} $$

.- Accuracy:

$$ \frac{TP+TN}{TP+FP+TN+FN} $$

.- Positive predictive value (PPV):

$$ \frac{TP}{TP+FP} $$

.- Negative predictive value (NPV):

$$ \frac{TN}{TN+FN} $$

.- Matthews Correlation Coefficient:

$$ \frac{TP\cdotp TN-FP\cdotp FN}{\sqrt{\left(TP+FN\right)\cdotp \left(TN+FP\right)\cdotp \left(TP+FP\right)\cdotp \left(TN+FN\right)}} $$

In all the previous equations: TP and FN are the numbers of correctly and incorrectly identified pathological variants, respectively; TN and FP are the numbers of correctly and incorrectly identified neutral variants, respectively.

The values of these parameters are provided in Additional files 5, 6: Tables S1, S2 (including also the corresponding TN, TP, FN, FP) for VS228; Additional file 7: Table S3, for VS2168. For simplicity, our analyses focus on the values of the MCC, but comparable results are obtained using accuracy (Additional files 8, 9, 10: Figures S3, S4, S5).

### External predictors

Application of the coincidence rule requires a minimum of two pathogenicity predictors. In our case we tried all possible combinations of the following five tools: PolyPhen-2 [30], SIFT [31], PON-P2 [32], MutationTaster2 [33] and CADD [34]. We chose them because their results are provided by software suites broadly used in the annotation of sequencing results in the clinical setting: SIFT, PolyPhen-2, CADD and MutationTaster2 are in ANNOVAR [35]; SIFT, PolyPhen-2 (after submission) and MutationTaster are in Alamut (http://www.interactive-biosoftware.com/doc/alamut-visual/2.9/missense-pred.html), SIFT and PolyPhen are included in Illumina’s Variant Studio software (http://support.illumina.com/downloads/variantstudio_userguide.html). PON-P2 is not included in none of them, but it was added because of its top-ranking performance relative to other predictors [15].

PolyPhen-2 (v2.2.2) was run locally with default parameters. SIFT and PON-P2 were run online (at http://sift.jcvi.org and http://structure.bmc.lu.se/PON-P2/, respectively). MutationTaster2 (http://www.mutationtaster.org) and CADD (http://cadd.gs.washington.edu) predictions were obtained using ANNOVAR [35]. The coverage of MutationTaster2, nor CADD tends to be lower than that of other methods because these two programs do not give predictions for amino acid substitutions resulting from more than one nucleotide change.

We also compared the performance of our method with that of two well-established consensus methods Condel [36] and CAROL [37]. We chose them because they build their consensus utilizing a minimum number of tools: Condel combines FATHMM [38] and MutationAssessor [39]; CAROL combines PolyPhen and SIFT. This makes them a good baseline for the performance of our approaches, which in their simpler form also combine two reference predictors. In the case of CONDEL the predictions were retrieved from the file ‘fannsdb.tsv.gz’, available for download at the website http://bg.upf.edu/fannsdb/. For CAROL run locally the R version of the program, downloaded from its website at the Sanger Institute (http://www.sanger.ac.uk/science/tools/carol).

## Results

Our goal is to test whether pathogenicity predictors with improved performance can be obtained for variants for which known methods do not agree in their predictions (these variants will be considered as pathogenic or neutral, depending on the method). The next two sections correspond to the two main steps followed to address this problem: (i) application of different versions of the coincidence rule for building the variant dataset; and (ii) development of the predictors. In a third and final section we describe what would be the overall state of the prediction problem, when considering together the cases that follow and the cases that break the coincidence rule.

NOTE. The results of this work apply to any single amino acid replacement, irrespective of whether it is the result of a single nucleotide change or not. These results remain essentially the same, except from minor variations that do not affect our conclusions, when we restrict our analyses to those variants resulting from a single nucleotide replacement only (Additional files 11, 12, 13, 14: Figures S6, S7, S8, S9).

### Applying the coincidence rule to build the variant dataset

To obtain the variant dataset for deriving our predictors we followed a simple protocol (Additional file 1: Figure S1) in which we first retrieved a total of 59,442 variants (15,723 pathogenic and 43,719 neutral variants, see Methods) distributed over a total of 228 proteins. Then, a set of known pathogenicity predictors was applied to these variants, keeping only those for which the predictors disagreed: these constituted our variant dataset, which we called PRDIS. Looking at this protocol, we see that each combination of pathogenicity predictors will give a different dataset. In this work, we have tried all possible combinations of five reference methods: SIFT, PolyPhen-2, CADD, PON-P2 and MutationTaster2. For example, for the case of two predictors, we produced a variant dataset for each of the following options: SIFT-PON-2, SIFT-PolyPhen-2, SIFT-CADD, SIFT-MutationTaster2, PON-P2-CADD, PON-P2-PolyPhen-2, etc. This resulted in a total of 26 PRDIS datasets.

**Fig. 1:**
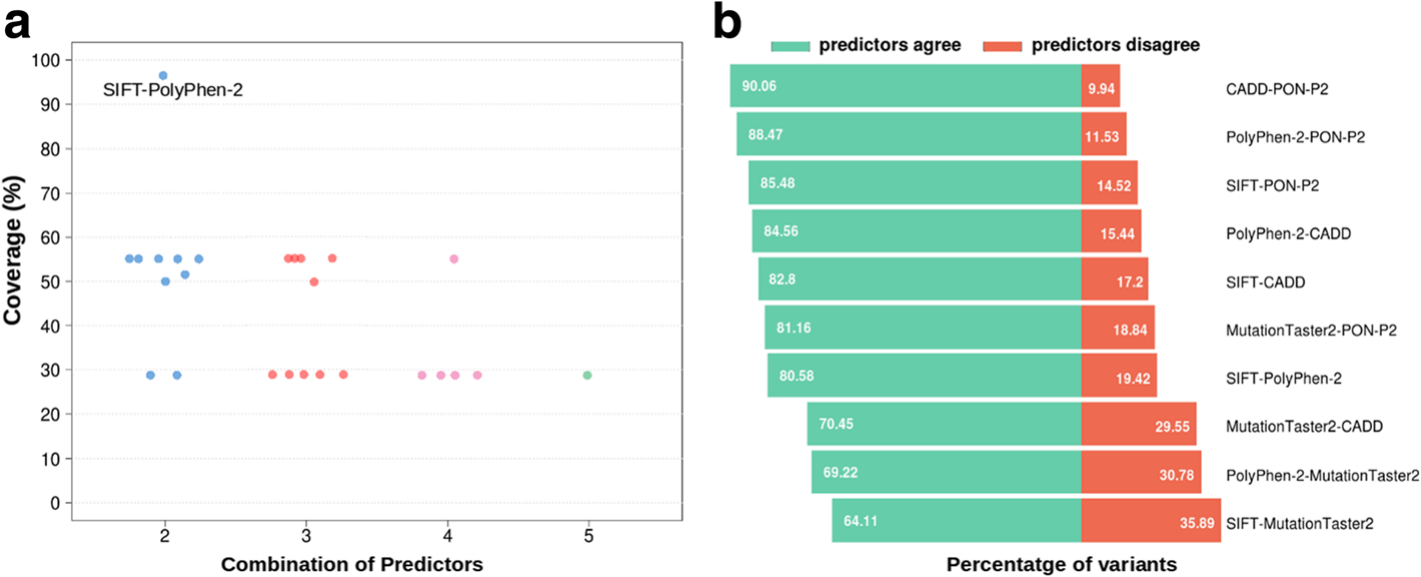
Statistics for the variant datasets in this study. **a** Percentage of cases that entered the study. The X-axis corresponds to the number of reference methods combined; each point corresponds to a specific combination of reference predictors (a slight offset is used for clarity purposes). **b** Composition of the PRDIS sets built from the combination of two reference predictors only. Each of the lines (percentage of agreements and disagreements to the left and right, respectively) corresponds to a point in (B), at x = 2

The first thing we observe during this process is that part of the 59,442 initial variants are discarded because predictions are not provided by all the methods for all variants (Fig. 1a; Additional file 5: Table S1). For example, there are only 57,349 (96% of the total of variants) instances for which the two predictors in the SIFT-PolyPhen (HDIV version) combination give an output; this number drops to 32,741 (55% of the total of variants) for the SIFT-MutationTaster2. These numbers reflect the original coverage of the reference methods. For example, SIFT, PolyPhen-2 (HDIV version) and MutationTaster2 generate results for 97%, 99% and 55% of the initial variants, respectively. It is then to be expected that the SIFT-PolyPhen-2 (HDIV version) combination gives more predictions than the SIFT-MutationTaster2 one. We also notice (Fig. 1b; Additional file 6: Table S2) that for those variants that pass the first step, there is an important percentage of cases for which predictors disagree, varying, for example, between 10% and 35% for the combinations of two predictors. For the remaining PRDIS datasets, the total number of mutations is large enough to suport the development of pathogenicity predictors; e.g. the combination of SIFT, PolyPhen (HDIV version), PON-P2, CADD and MutationTaster2 gives a PRDIS set with 5815 variants. As a reference, protein-specific predictors have been developed with variant datasets with 50/50 neutral/pathogenic instances [15].

**Fig. 2:**
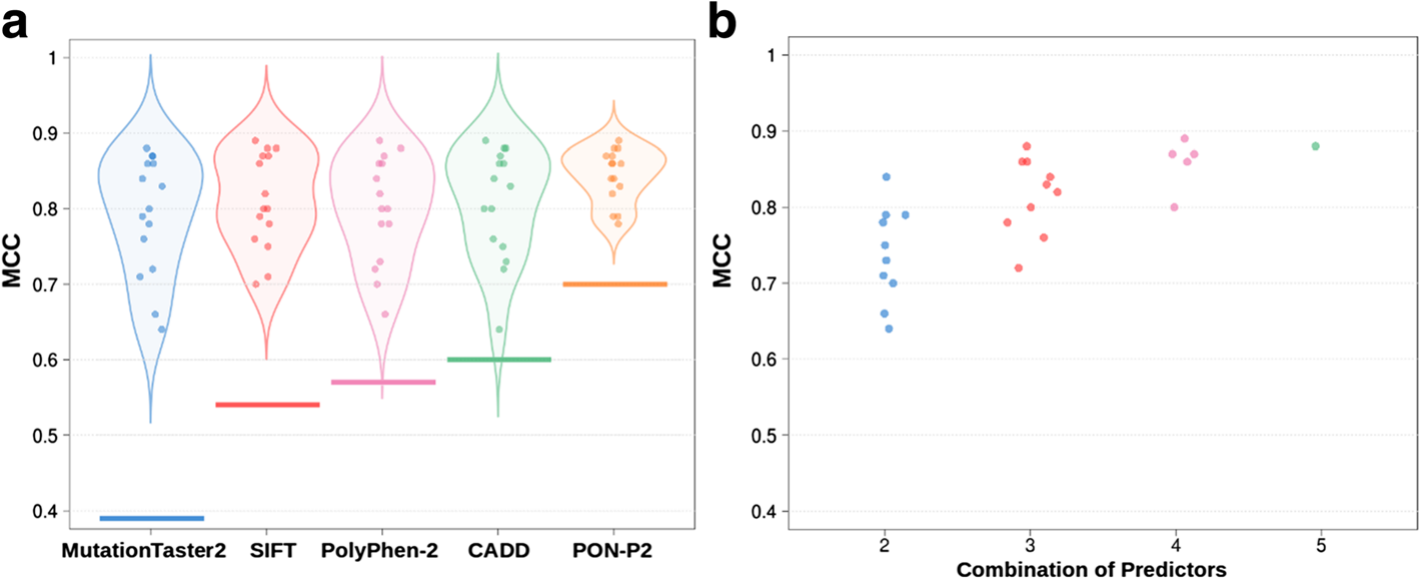
Success rate of predictions obtained following the coincidence rule. In the coincidence rule (see main text) computational information is accepted as supporting evidence in clinical settings only when the pathogenicity predictions of different methods agree. Here we describe how the success rate of this rule depends on the chosen in silico predictors. **a** Violin plots for the Matthews Correlation Coefficients (MCC) grouped by method. Each violin plot corresponds to all possible combinations of reference predictors that include the method shown at the bottom. For example, the first plot to the left represents all combinations of five reference predictors (SIFT, PolyPhen-2, PON-P2, CADD and MutationTaster2) that include MutationTaster2. The thick lines at the bottom of each violin plot represent the individual performance of the reference predictors. **b** Dependence of MCC values on the number of predictors used to implement the coincidence rule

We checked the success rate of the coincidence rule for the variants for which the combined predictors agreed (Additional file 1: Figure S1). We found that using this rule always gives better results than using the predictors alone (Fig. 2a): it has the ability to select the subset of predictions, from a given method, that are more accurately predicted. For example, in the case of PolyPhen-2 (HDIV version) the individual MCC is 0.57, while that of the SIFT-PolyPhen-2 (HDIV version) is 0.70. For PON-P2 the individual MCC is 0.70, while that of its combination with MutationTaster2 is 0.79. We also see that increasing the number of methods results in better success rates, although the trend is asymptotic (Fig. 2b).

### Building specific tools for the variants with discordant predictions (PRDIS)

This section is divided into four subsections. In the first one (“Obtaining predictors...”), we show that we can obtain non-random predictors for variants in PRDIS. The remaining three subsections (“Can specific predictors outperform reference...”; “Can specific predictors outperform simple...”; “Testing the reach...”) are devoted to compare the performance of these specific predictors with that of (i) reference tools (PolyPhen-2, SIFT, PON-P2, CADD and MutationTaster2), (ii) with that of consensus tools (Condel and CAROL), and (iii) extending the main conclusion to proteins outside VS228.

### Obtaining specific predictors for PRDIS

For each PRDIS dataset we derived a set of four specific predictors (Additional file 2: Figure S2). They correspond to the different combinations of the following options: two possible inputs and two models of different complexity. The two inputs were: (i) a simple one, having only the prediction scores from the reference methods; and (ii) and extended version of the simple input augmented with three additional properties (Blosum62 matrix elements, Shannon’s entropy and the position-specific scoring matrix elements). The two complexity levels for the models were: a neural network with no hidden layers and one with one hidden layer and two nodes. The performance figures are the average of 10 replicas of the 5-fold cross validation process, to smooth out fluctuations.

**Fig. 3:**
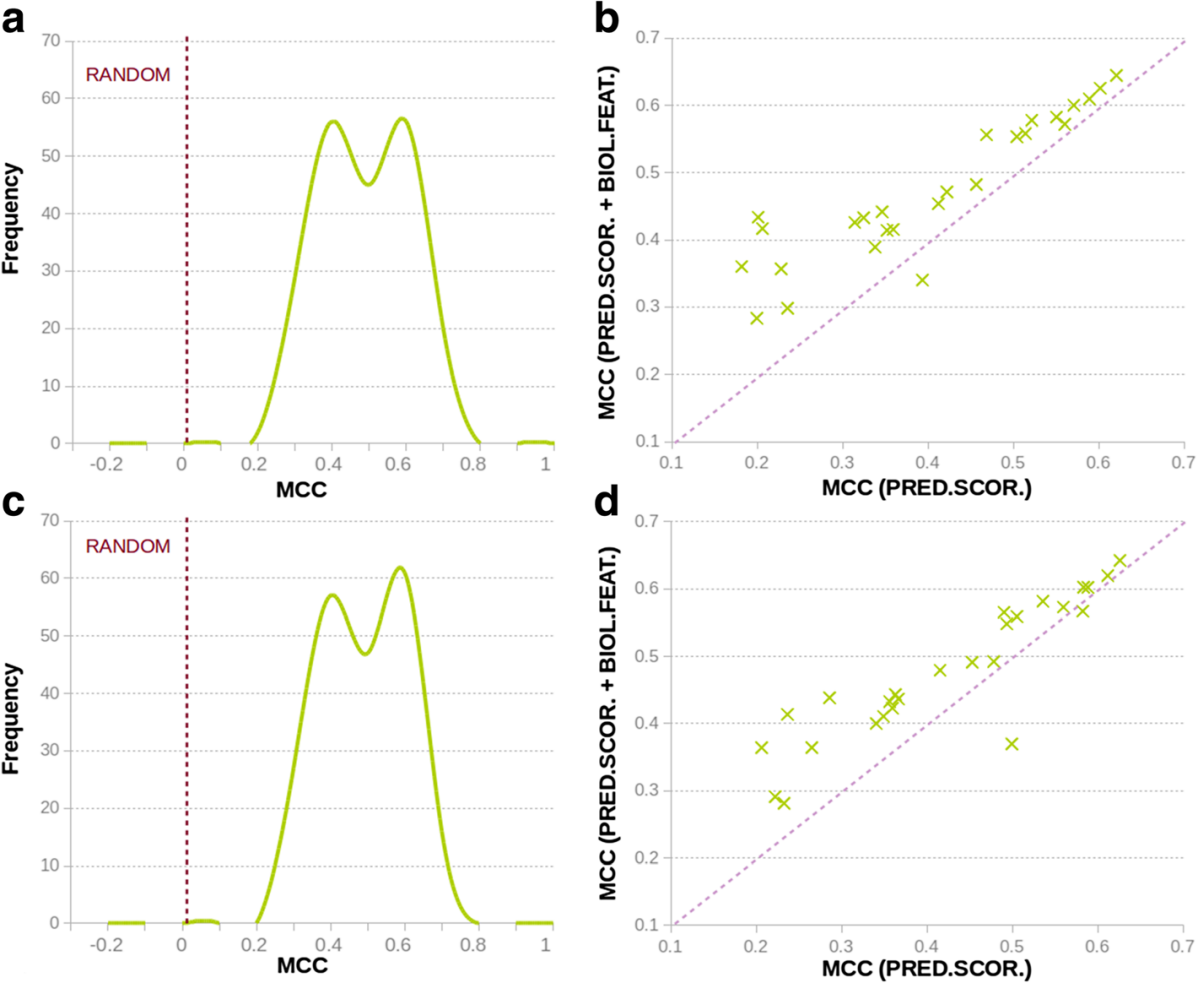
Performance of the PRDIS specific predictors. **a** and **c**. Frequency distribution of MCC values for all the specific predictors generated in this work: (**a**) data for simple neural networks; (**c**) data for neural networks with one hidden layer and two nodes. Shown with a dashed line is 0, the MCC value for a random predictor. We see that specific predictors are systematically better than the random predictor. **b** and **d**. Contribution of the three biochemical/biophysical properties (Blosum62 elements, Shannon’s entropy and Position specific scoring matrix elements; see Materials and Methods) to improve the performance of the specific predictors. Points above the dotted line correspond to cases where use of these properties improves the performance of a specific predictor. We see that this is essentially always the case. **b** and **d** correspond to the simpler and to the one hidden layer neural networks, respectively

Our results show (Fig. 3) that the vast majority of the specific predictors have performances above those of a random method. That is, there is a signal in PRDIS allowing the discrimination between pathogenic and neutral mutations; this signal can be recognized with the variant features employed in this work.

We also observe bimodality in the MCC distributions (Fig. 3a and c); the peaks at high and low MCC values predominantly correspond to methods using the extended and the reduced inputs, respectively. This is in agreement with our previous experience where the use of biochemical/biophysical features allowed us to resolve a contradiction between SIFT and PolyPhen-2 predictions for variant F367 V in FOXP3 [40].

These results remain essentially unchanged whether we use the simple (Fig. 3a and b) or the complex neural network model (Fig. 3c and b).

### Can specific predictors outperform reference (SIFT, PolyPhen-2, PON-P2, CADD, MutationTaster2) methods?

**Fig. 4:**
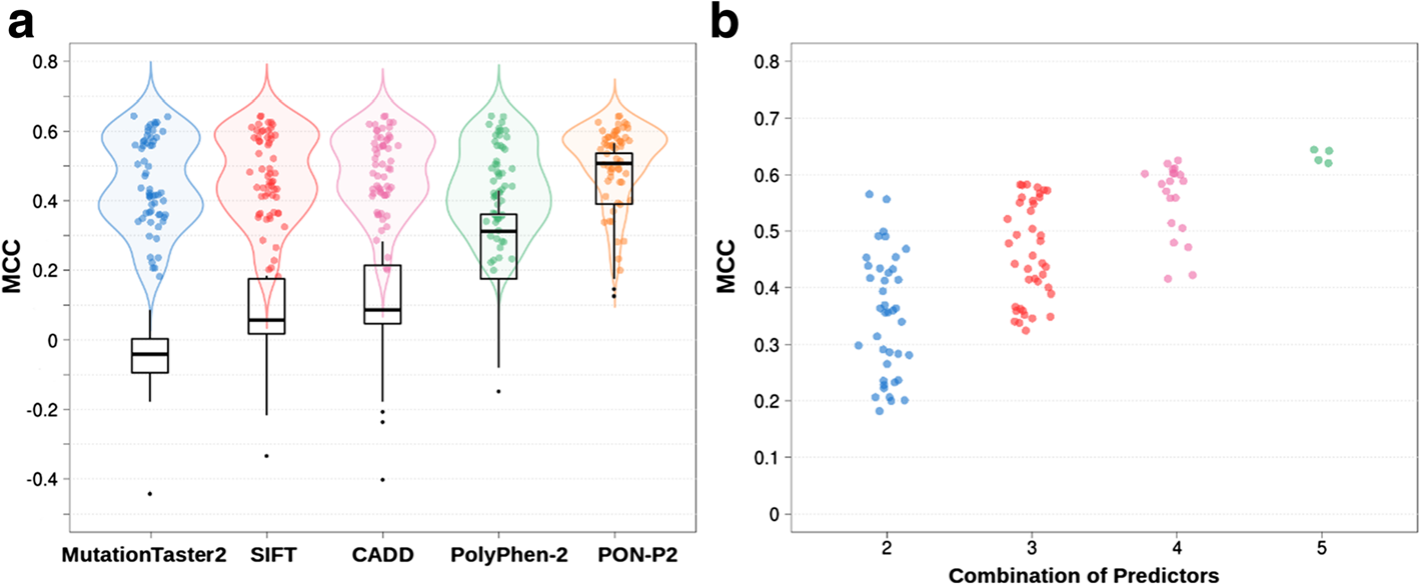
The contribution of reference methods to PRDIS specific predictors. In (**a**) we compare the performance of PRDIS specific methods, represented with violin plots with that of the reference methods (SIFT, PolyPhen-2, PON-P2, CADD and MutationTaster2), represented with black boxplots. We see that specific methods are frequently better than reference methods, but there is an increasing overlap between both approaches as the performance of the reference method grows (e.g. in the cases of PON-P2 or PolyPhen-2). **b** Performance depends on the number of reference predictors combined: the more we use, the more likely we are to obtain higher performances

In Fig. 4, for each reference predictor (SIFT, PON-P2, etc) we plot both its performance (MCC) distribution (black boxplot) and that of the specific predictors that include its score among their input attributes (color violin plot). The first thing we notice is that here the performance of the reference predictors is lower than for the case of variants with concordant predictions (Fig. 2a). The same happens when we consider specific tools instead. For these, the upper-bounds of the MCC distributions are between 0.6 and 0.7 (Fig. 4b), while for the consistency rule MCCs values can reach 0.9 (Fig. 2b). With the lower-bounds we see a similar effect. For example, for specific predictors involving MutationTaster2 and CADD the lower-bounds are around 0.2; for applications of the consistency rule involving these two predictors, the values are above 0.6. Overall, this indicates that the problem of discriminating between neutral and pathogenic variants is harder for PRDIS than for non-PRDIS variants.

We also observe how the performances (MCC) of the reference and specific predictors are related. In particular, we see that when the success rate of the reference predictor is high, the same happens with that of the derived specific predictors. For PON-P2, the method with the highest success rates in this work, specific’s MCCs concentrate near 0.65; for the next performer, PolyPhen-2 (HDIV version), specific’s MCCs show a shift towards lower values; and so on. We also find that as the individual performances of the reference methods drop (when the black boxplots move towards 0 in Fig. 4a) the difference between specific and reference predictors grows. In summary, the better the performance of the reference method is, the more it resembles that of its related specific predictors.

We have seen that prediction of PRDIS variants is a hard problem and that specific tools provide a promising approach to its solution. In this context, a natural question is: has the performance of specific tools in PRDIS variants (Additional file 6: Table S2) reached that of reference methods in average variants (Additional file 5: Table S1)? Our results indicate that, in general, this is not yet the case. The differences between reference methods do not generally alter this conclusion; there is, however, some variability that depends on the parameters considered. More precisely, for MCC we see that SIFT is above 39 out of 57 (68%) specific predictors, PolyPhen-2 (HDIV version) is above 75%, CADD is above 88%, and PON-P2 is above 100%. If we turn to variant-specific parameters, like sensitivity (pathogenic) and specificity (neutral) we find that for sensitivity, all reference methods are above all specific predictors, except for SIFT, which is above 72% of them. For specificity, the situation is somewhat reversed. The specificity of MutationTaster2 for the average variant, 0.47, is below that of all specific tools in PRDIS variants; in our dataset, this method shows a prediction bias towards pathogenicity. This bias is also present in the other reference methods, which show specificities below their sensitivities. However, the difference with specific methods becomes gradually smaller, from PolyPhen-2 (HDIV version), which is above 14%, to PON-P2, which is above 56%. The other variant-related parameters (PPV and NPV) are also of interest; however, they have a high dependency on the sample composition that makes difficult the comparison. Having said that, for PPV we see that reference methods, when applied to the average variant, outperform specific methods, when applied to PRDIS variants, in different degrees: MutationTaster2 is above 39%, PolyPhen-2 (HDIV version) is above 70%, SIFT is above 77%, CADD is above 86%, and PON-P2 is above 100%. Given that the sample effect is unclear in this case, we also give (Additional file 15: Figure S10) the comparison of PPV values when applying to PRDIS variants both reference and specific methods. We find that the latter clearly outperforms the former. On the basis of both results we believe that for PPV there is a complementary situation where both approaches mutually outperform each other; however, we cannot go any further, given the sample differences. In summary, the overall view is that the performance of specific methods in the hard problem of PRDIS variants has not yet reached that of reference methods in the problem of average variants. Consequently, the success rates of specific methods are still below the levels above which bioinformatics evidence is considered as supporting evidence in the clinical setting [11].

**Fig. 5:**
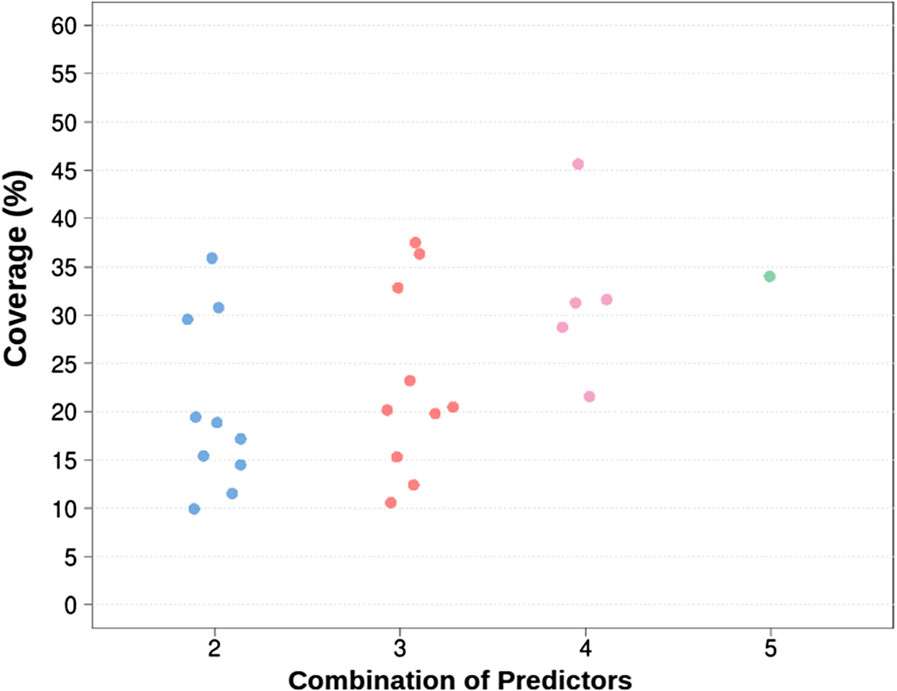
Coverage of the specific predictors. The number of variants used to obtain specific predictors grows as we increase the number of standard methods used to build the PRDIS set. This is to be expected, since the more methods we use, the easier it is to find a discordance between predictions

It must be noted that the size of the PRDIS sets varies gradually, increasing as we add more predictors (Fig. 5).

### Can specific predictors outperform simple consensus (Condel, CAROL) methods?

As we have seen before, our specific predictors are obtained using as input the score of reference predictors (enriched, in some cases, with other features). In this sense they are similar to consensus methods [12, 13, 37, 41], which also use the output of known predictors as their input. Here we compare PRDIS specific predictors with Condel and CAROL. These two methods constitute an interesting reference since, in spite of their good performance, they are technically simple: they utilize a minimum number of known predictors to build their consensus, (MutationAssessor, FATHMM) for Condel and (PolyPhen, SIFT) for CAROL.

**Fig. 6:**
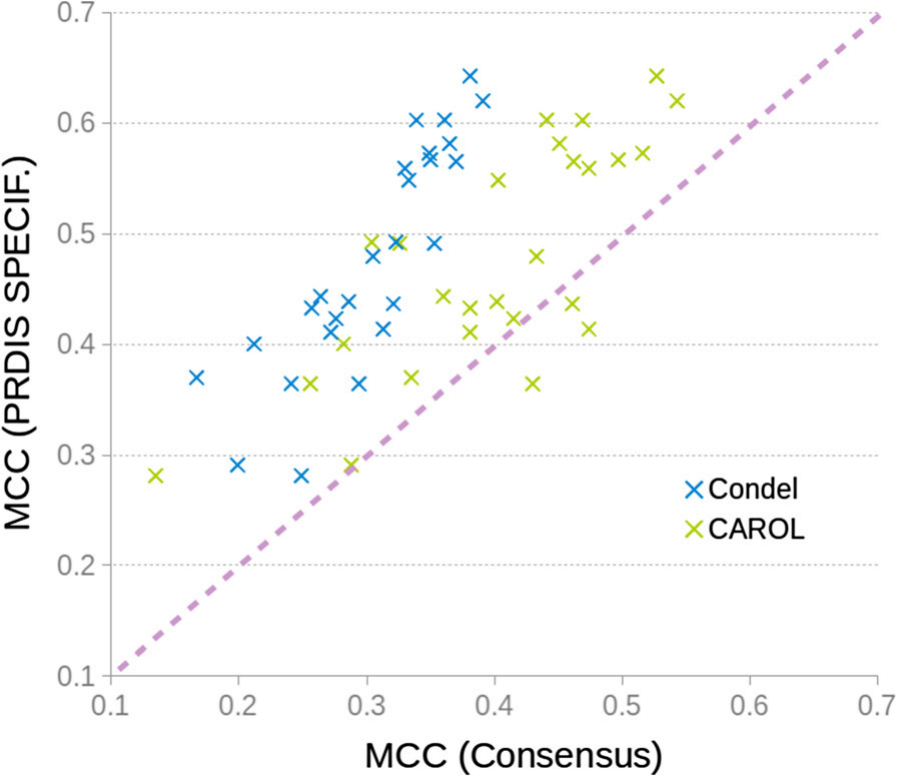
Comparison between the performance of PRDIS specific and conventional consensus predictors. We represent the MCC of PRDIS specific predictors (Y-axis) against that of conventional consensus methods (Condel and CAROL; X-axis). Points above the diagonal indicate that the former tend to outperform the latter, for PRDIS variants. We see that this is generally the case, although with a trend in the performance of CAROL predictions to reach the level of specific methods

We see (Fig. 6) that PRDIS specific predictors outperform always Condel and almost always CAROL. This indicates that using PRDIS data for developing specific predictors is a good option relative to the technically simple (but powerful) predictors such as Condel and CAROL.

### Testing the reach of the specific approach

The specific approach presented here is based on identifying the variants that do not follow the coincidence rule and train predictors specific for them. In Results section "Can specific predictors outperform reference (SIFT, PolyPhen-2, PON-P2, CADD, MutationTaster2) methods?" we have seen (Fig. 4), using a standard cross-validation scheme, that this approach generally outperforms reference predictors (PolyPhen-2, SIFT, etc.) for variants in VS228. To test if this conclusion also holds for proteins not represented in VS228, we applied our specific models to PRDIS sets obtained from VS2168. It is important to note that VS228 and VS2168 contain variants from different proteins. That is, proteins contribute variants either to one set or the other, but not to both.

**Fig. 7:**
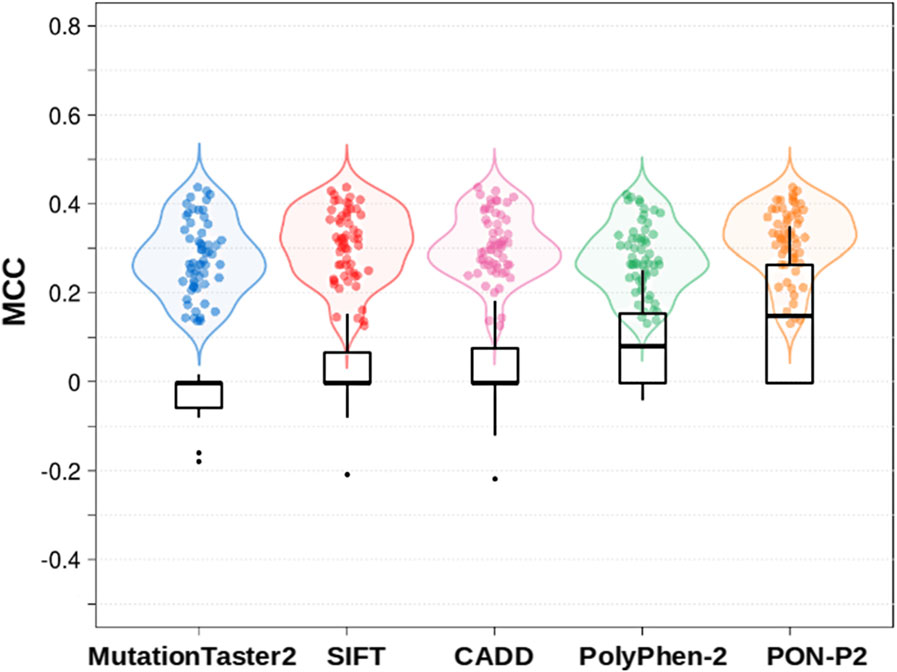
The relationship between PRDIS specific predictors and reference methods for proteins in VS2168 dataset. In this figure we compare the performance of PRDIS specific methods when applied to the variants in VS2168. None of the proteins represented in this set contributes a variant to VS228, which is the dataset used to train the specific predictors and obtain a cross-validated estimate of their performance (Fig. 4). The MCCs of the specific methods are represented with violin plots and those of the reference methods (SIFT, PolyPhen-2, PON-P2, CADD and MutationTaster2) are represented with black boxplots. We see that, in spite an overall decrease in performance for all tools displayed, specific methods are frequently better than reference methods, but there is an increasing overlap between both approaches as the performance of the reference method grows (e.g. in the cases of PON-P2 or PolyPhen-2). This result confirms the conclusion obtained with the VS228 set (Fig. 4)

In Fig. 7, which is analogous to Fig. 4, for each reference predictor (SIFT, PON-P2, etc.) we plot both its performance distribution (black boxplot) and that of the specific predictors that include its score among their input attributes (color violin plot). We see that, apart from an overall trend towards lower success rates, the results are comparable to those obtained for VS228: specific predictors tend to outperform reference predictors and, as the performance of the latter improves, the difference between approaches decreases.

Given that the overall success rates for VS2168 have decreased, these results do not affect the previous observation according to which the performance of reference methods applied to the average variant is higher than that of specific predictors applied to PRDIS variants.

### How good is the combination of the coincidence rule and PRDIS specific predictors?

**Fig. 8:**
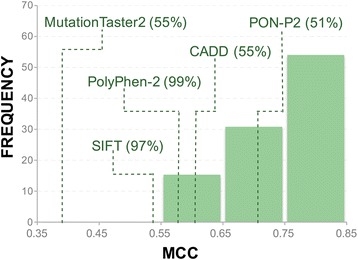
Performance of the hybrid predictor. For each possible hybrid predictor in this work, we computed its MCC. In the figure we show the frequency histogram of these values. With dashed lines we show the prediction of the different reference methods, estimated on the original set of 59,442 variants. We see that hybrid methods tend to outperform reference methods, although this depends on the latter. For example, PON-P2 alone is better than many of the hybrids

Combined use of the coincidence rule and PRDIS specific predictors results in a hybrid method that can produce predictions for the major part of the variant dataset (Additional file 16: Figure S11). There is one hybrid method for each combination of reference predictors; for example, when our reference methods are SIFT and PolyPhen-2, we will have one associated coincidence rule and one PRDIS specific predictor. We see (Fig. 8) that, when applying the hybrid approach to the original dataset of variants, most of the hybrid methods have performances higher than those of the reference methods (estimated on the same dataset). For example, MutationTaster2 is outperformed by all hybrid methods while, at the other end of the scale, PON-P2 outperforms 50% of the hybrid methods. This is not related to coverage (percentage of variants predicted), since both MutationTaster2 and PON-P2 have very similar values, 55% and 51%, respectively. Detailed performance results are provided in Additional file 17: Table S4.

## Discussion

In the last years the use of computational evidence for the identification of pathogenic sequence variants in the clinical setting is being gradually reconsidered [6–8]. However, given their still limited accuracy, the unrestricted use of pathogenicity predictors is not advised [42]. This idea has taken a more precise shape in the ACMG/AMP guidelines for variant interpretation [11], where computational results are considered as supporting evidence only when the tools used to generate them agree (what we call consistency rule in this work). Otherwise, computational data are rejected. Seeking agreement between methods is a natural approach to enhance our prediction ability and is particularly valuable when several (partial) solutions to the same problem are available [43]. For the case of pathogenicity predictions this approach has also been tried. For example, using a small set proteins Chan et al. [44] find that taking the consensus of four prediction tools (naive use of Blosum62, SIFT, PolyPhen and A-GVGD) results in an increased predictive value, although at the price of a substantial reduction in the number of predictions. In general, it is accepted that this approach may produce detectable improvements over the use of single methods [10, 12, 13, 44], although combining tools may have its problems [12]. In our case, we observe that simple application of the consistency rule to our variant dataset (Additional file 1: Figure S1) also results in high success rates (Fig. 2b), better than those of the reference methods employed to implement the rule. However, there is a percentage of cases -considered to be hard to predict by Capriotti et al. [13], for which reference predictors disagree and consequently computational evidence should be discarded in a medical environment [11]. These cases represent about 10% to 45% of the total number of variants (Additional file 5: Table S1) and their prediction constitutes the main goal of our work. In particular, we have explored whether by focusing our efforts on these cases we can derive specific predictors outperforming known methods. We have tested this idea on VS228, a set of 59,442 variants spread over 228 proteins of medical interest. To this end we have trained a series of neural network predictors (Additional file 2: Figure S2), trying two different inputs and two different complexity levels, and estimated their performance using a 5-fold cross-validation procedure. Our results indicate that indeed using this specific approach gives tools with increased success rates, which are better than those of the reference (Fig. 4; SIFT, PolyPhen-2, PON-P2, CADD and MutationTaster2) and consensus (Fig. 6; Condel, CAROL) methods considered.

We also observe that the overall performance of PRDIS specific tools (Fig. 4a) is still below that obtained for variants for which predictors agree in their predictions (Fig. 2a). This reflects the gap described by Capriotti et al., [13] between easier and harder cases. However, the simplicity of our models suggests that there is still room for the development of models that can close this gap. And, even at this early stage, specific tools can already be useful. For example, let us consider the following variants: Y482C, in ATP-binding cassette sub-family A member 1, which causes High-density lipoprotein deficiency; Y72C, in Hypoxanthine-guanine phosphoribosyltransferase, which causes hyperuricaemia and chronic tophaceous gout, and W453R, in Cytochrome b-245 heavy chain, which causes X-linked Chronic Granulomatous disease. The three variants are correctly predicted by SIFT, PON-P2, CADD and MutationTaster2, but are missed by PolyPhen-2 (HDIV version). Our specific method that uses the five scores as input features correctly identifies the variants as pathogenic (scores: 0.67, 0.62 and 0.57; all above 0.5). In addition, if our tool also includes the three biological features as part of the input, the reliability of the predictions is higher (scores: 0.82, 0.92 and 0.77; all above 0.5). Apart from showing the potential of the PRDIS specific predictors, this example can be used to understand why sometimes predictions by PolyPhen-2 are in contradiction with those from the other methods. A detailed analysis of PolyPhen-2’s MSAs shows that, for the three variants considered, the pathogenic amino acid appears once in the column of the mutated amino acid, in a non-human species: for Y482C the cysteine is present in *S. harrisii*, for Y72C the cysteine is present in *P. tricornutum*, and in W453R the tryptophan appears in *R. norvegicus*. Since the score of PolyPhen-2 takes into account this fact, this could explain the deviating prediction. We reran PolyPhen-2 after eliminating the affected sequences from the MSAs and the three variants were now correctly predicted as pathological, in accordance with the other reference methods. We had previously found a similar situation in a FOXP3 variant, when integrating PolyPhen-2, SIFT and structural evidence [40].

We have extended the validity of our principal conclusion applying our trained predictors to the 322,270 variants in VS2168, which are distributed over 2168 proteins not represented in VS228. Our results (Fig. 7) indicate that, in spite an overall decrease in success rate, the main conclusion of this work holds: specific predictors tend to outperform reference methods.

### Partitioning the variant space and focusing on the hardest problems

Methodologically, the approach presented is based on the idea of partitioning the dataset of variants according to a given criterion and then derive a specific predictor for some, or for all, of the resulting subsets. The underlying rationale is that the partitioning step may give improved prediction tools either because the resulting subsets are more homogeneous or because it allows us to put our efforts on tackling the more difficult parts of the prediction problem. The development of protein-specific predictors [15] corresponds to the first situation: every subset is constituted by variants from a single protein. The specific predictors show good performances relative to non-specific methods (e.g. PolyPhen-2, CADD, etc) although not always (in many cases PON-P2 outperforms the protein-specific methods). This may be due to different factors, for example the new prediction problem defined by the data in the protein-specific subset may require also an adaptation of the model, e.g. including specific terms for the protein. This is for example what has been recently done by [45] for KinMutRF, their pathogenicity predictor for kinases; in this tool the authors employ kinase-specific features in their input, such as specific Gene Ontology terms. Our work corresponds to the second case, in which partitioning through application of the coincidence rule separates variants “easy” to predict from those that are harder to predict, which are those for which known methods disagree (PRDIS in our case). This difficulty gap has been already mentioned by Capriotti et al. [13] who describe how their consensus predictor Meta-SNP performs much better for those cases for which their four constituting predictors PANTHER, PhD-SNP, SIFT and SNAP agreed in their verdict than for those where they disagreed. Here we have shown that developing specific predictors for this hard case benefits our performance for PRDIS and improves overall prediction performance (Fig. 8). It is worth noting, however, that improvement size varies depending on the performance of the reference predictors, a trend already observed in the case of protein-specific predictors. That is, when the performance of the reference predictor is high (e.g. like in the case of PON-P2), it is more difficult to obtain outperforming specific predictors (Fig. 4).

## Conclusions

In the clinical setting, the use of computational evidence on variant pathogenicity is restricted to those cases where there is a full coincidence between in silico tools (see ACMG/AMP guidelines [11]). This coincidence rule results in a loss of information for a percentage of variants that varies between 10% and 35%, when combining two predictors. In this work, we have focused on the development of specific tools for these variants and on testing whether we can obtain better success rates than known methods. We find that this is indeed the case, although some existing methods (PON-P2 and PolyPhen-2) already give a competitive performance (with varying coverages) that is more difficult to improve.

## Additional files

Additional file 1: Figure S1. Obtention of the variant datasets. The figure shows how we obtained the subsets of variants for which pathogenicity predictors disagreed (PRDIS, within the red contour) and agreed (within the blue contour), respectively. For a certain percentage of cases, some predictors would not give a prediction for the variables (indicated as “No output for predictor(s)”). The original set of protein sequence variants was obtained from (see Materials and Methods): (i) UniProt database, for pathogenic variants; (ii) a homology-based model, for neutral variants. (PNG 673 kb)

Additional file 2: Figure S2. Obtention of specific predictors for PRDIS variants. For each combination of the five reference methods used in this work (SIFT, PolyPhen-2, PON-P2, CADD and MutationTaster2) we obtained PRDIS, the subset of those variants for which the reference predictors disagreed. Then, for each of these PRDIS sets, we produced four different predictors, which differed either in the neural network model or in the neural network input. For the neural network model we tried two options: (i) no hidden layers (NN: 0); and (ii) one hidden with two nodes (NN: 2). For the neural network inputs, we tried two options: (i) the scores of the reference predictors; and (ii) the scores of the reference predictors enriched with three biological features (Blosum62 matrix elements, Shannon’s entropy, Position-specific scoring matrix elements; see Materials and Methods). Boxed in red is the case where PRDIS was obtained using SIFT and PolyPhen-2 as reference methods. (PNG 666 kb)

Additional file 3: Pathogenic variants. Each line corresponds to a variant, providing: the amino acid replacement and its location in the protein sequence, the UniProt code for the protein, the values of the contribution of the three biochemical/biophysical properties (Blosum62 elements, position specific scoring matrix elements and Shannon’s entropy) followed by the output of the pathogenicity predictions for the reference methods used in this work (for PolyPhen-2 we give the output of its two versions –HDIV and HVAR- although in this work we only used HDIV predictions), and ‘?’ is given when no output was provided by the method. The last column gives the dataset where the variant belongs, either VS228 or VS2168. (CSV 1616 kb)

Additional file 4: Neutral variants. Each line corresponds to a variant, providing: the amino acid replacement and its location in the protein sequence, the UniProt code for the protein, the values of the contribution of the three biochemical/biophysical properties (Blosum62 elements, position specific scoring matrix elements and Shannon’s entropy) followed by the output of the pathogenicity predictions for the reference methods used in this work (for PolyPhen-2 we give the output of its two versions –HDIV and HVAR- although in this work we only used HDIV predictions), and ‘?’ is given when no output was provided by the method. The last column gives the dataset where the variant belongs, either VS228 or VS2168. (ZIP 5531 kb)

Additional file 5: Table S1. Success rate of the coincidence rule, for the all the different combinations of reference predictors (SIFT, PolyPhen-2, PON-P2, CADD and MutationTaster2). The performance measures are the six standard measures (MCC, accuracy, sensitivity, specificity, PPV and NPV) described in the Materials and Methods section. We give: the raw TP, TN, FP and FN values; the coverage relative to the original dataset of 59,442 variants (VS228) and the number of cases where the predictors coincide. (PDF 28 kb)

Additional file 6: Table S2. Prediction performance for the PRDIS specific predictors in this work for VS228; each corresponds to a different combination of the reference predictors (SIFT, PolyPhen-2, PON-P2, CADD and MutationTaster2). The performance measures are the six standard measures (MCC, accuracy, sensitivity, specificity, PPV and NPV) described in the Materials and Methods section. We also give: the total number and the percentage of cases, and the raw TP, TN, FP and FN values. (PDF 26 kb)

Additional file 7: Table S3. Prediction performance for the PRDIS specific predictors in this work for VS2168 dataset; each corresponds to a different combination of the reference predictors (SIFT, PolyPhen-2, PON-P2, CADD and MutationTaster2). The performance measures are the six standard measures (MCC, accuracy, sensitivity, specificity, PPV and NPV) described in the Materials and Methods section. We also give: the total number and the percentage of cases, and the raw TP, TN, FP and FN values. (PDF 26 kb)

Additional file 8: Figure S3. In the coincidence rule (see main text) computational information is accepted as supporting evidence in clinical settings only when the pathogenicity predictions of different methods agree. Here we describe how the success rate of this rule depends on the chosen in silico predictors. (A) Violin plots for the Accuracy grouped by method. Each violin plot corresponds to all possible combinations of reference predictors that include the method shown at the bottom. For example, the first plot to the left represents all combinations of five reference predictors (SIFT, PolyPhen-2, PON-P2, CADD and Mutation Taster2) that include MutationTaster2. (B) Dependence of Accuracy values on the number of predictors used to implement the coincidence rule. (PNG 135 kb)

Additional file 9: Figure S4. (A) and (C). Frequency distribution of accuracy values for all the specific predictors generated in this work: (A) data for simple neural networks; (C) data for neural networks with one hidden layer and two nodes. Shown with a dashed line is 0.5, the accuracy value for a random predictor. We see that specific predictors are systematically better than the random predictor. (B) and (D). Contribution of the three biochemical/biophysical properties (Blosum62 elements, Shannon’s entropy and Position specific scoring matrix elements; see Materials and Methods) to improve the performance of the specific predictors. Points above the dotted line correspond to cases where use of these properties improves the performance of a specific predictor. We see that this is essentially always the case. (B) and (D) correspond to the simpler and to the one hidden layer neural networks, respectively. (PNG 194 kb)

Additional file 10: Figure S5. In (A) we compare the performance of PRDIS specific methods, represented with violin plots with that of the reference methods (SIFT, PolyPhen-2, PON-P2, CADD and Mutation Taster2), represented with black boxplots. We see that specific methods are frequently better than reference methods, but there is an increasing overlap between both approaches as the performance of the reference method grows (e.g. in the cases of PON-P2 or PolyPhen-2). (B) Performance depends on the number of reference predictors used: the more predictors are used, the more likely to obtain higher performances. (PNG 219 kb)

Additional file 11: Figure S6. The results in this figure are computed for the subset of amino acid variants resulting from single nucleotide replacements only. (A) Percentage of cases that entered the study. The X-axis corresponds to the number of reference methods combined; each point corresponds to a specific combination of reference predictors (a slight offset is used for clarity purposes). (B) Composition of the PRDIS sets built from the combination of two reference predictors only. Each of the lines (percentage of agreements and disagreements to the left and right, respectively) corresponds to a point in (B), at x = 2. (PNG 115 kb)

Additional file 12: Figure S7. The results in this figure are computed for the subset of amino acid variants resulting from single nucleotide replacements only. In the coincidence rule (see main text) computational information is accepted as supporting evidence in clinical settings only when the pathogenicity predictions of different methods agree. Here we describe how the success rate of this rule depends on the chosen in silico predictors. (A) Violin plots for the Matthews Correlation Coefficients (MCC) grouped by method. Each violin plot corresponds to all possible combinations of reference predictors that include the method shown at the bottom. For example, the first plot to the left represents all combinations of five reference predictors (SIFT, PolyPhen-2, PON-P2, CADD and MutationTaster2) that include MutationTaster2. (B) Dependence of MCC values on the number of predictors used to implement the coincidence rule. (PNG 113 kb)

Additional file 13: Figure S8. The results in this figure are computed for the subset of amino acid variants resulting from single nucleotide replacements only. (A) and (C). Frequency distribution of MCC values for all the specific predictors generated in this work: (A) data for simple neural networks; (C) data for neural networks with one hidden layer and two nodes. Shown with a dashed line is 0, the MCC value for a random predictor. We see that specific predictors are systematically better than the random predictor. (B) and (D). Contribution of the three biochemical/biophysical properties (Blosum62 elements, Shannon’s entropy and Position specific scoring matrix elements; see Materials and Methods) to improve the performance of the specific predictors. Points above the dotted line correspond to cases where use of these properties improves the performance of a specific predictor. We see that this is essentially always the case. (B) and (D) correspond to the simpler and to the one hidden layer neural networks, respectively. (PNG 172 kb)

Additional file 14: Figure S9. The results in this figure are computed for the subset of amino acid variants resulting from single nucleotide replacements only. In (A) we compare the performance of PRDIS specific methods, represented with violin plots with that of the reference methods (SIFT, PolyPhen-2, PON-P2, CADD and MutationTaster2), represented with black boxplots. We see that specific methods are frequently better than reference methods, but there is an increasing overlap between both approaches as the performance of the reference method grows (e.g. in the cases of PON-P2 or PolyPhen-2). (B) Performance depends on the number of reference predictors combined: the more we use, the more likely we are to obtain higher performances. (PNG 258 kb)

Additional file 15: Figure S10. Comparison between PPV values for PRDIS specific and reference predictors. The figure shows that combination of reference methods (specific predictors) gives better PPV than reference methods alone: for only seven cases the reference approach outperformed the specific approach. (PNG 68 kb)

Additional file 16: Figure S11. A hybrid predictor. A hybrid method is implicitly defined if the coincidence rule is used as a pre-classification step. In this method, the variants for which standard methods agree will be assigned this coinciding prediction; for PRDIS variants, a prediction will be obtained from the PRDIS specific method. The final performance of this hybrid method is obtained by combining that of the two cases. (PNG 607 kb)

Additional file 17: Table S4. Prediction performance for the hybrid predictor. We give the raw TP, TN, FP and FN values and the values of the six standard measures (MCC, accuracy, sensitivity, specificity, PPV and NPV) described in the Materials and Methods section. (PDF 24 kb)

## Abbreviations

MCC: Matthews Correlation Coefficient
PPV and NPV: positive and negative predictive values.
PRDIS: set of variants for which predictions from known methods disagree
TN and FP: number of correctly and incorrectly identified neutral variants
TP and FN: number of correctly and incorrectly identified pathological variants, respectively
