# Supplementary figures and images for: Development of pathogenicity predictors specific for variants that do not comply with clinical guidelines for the use of computational evidence
Source: BMC Genomics. 2017 Aug 11;18(Suppl 5):569. doi: 10.1186/s12864-017-3914-0 (PMC5558188; doi:10.1186/s12864-017-3914-0)

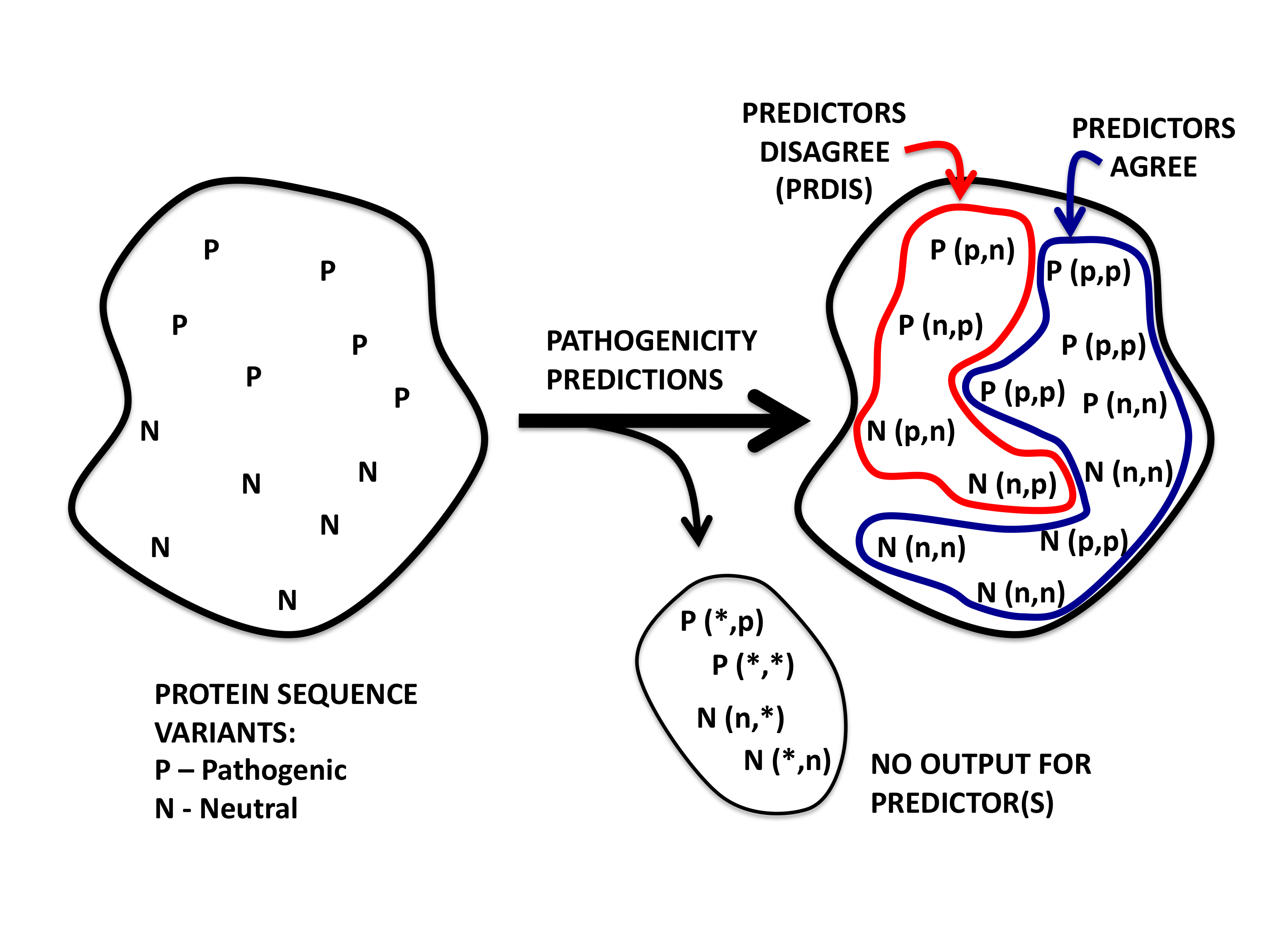

Supplement: Supplementary file 1 — Obtention of the variant datasets. The figure shows how we obtained the subsets of variants for which pathogenicity predictors disagreed (PRDIS, within the red contour) and agreed (within the blue contour), respectively. For a certain percentage of cases, some predictors would not give a prediction for the variables (indicated as “No output for predictor(s)”). The original set of protein sequence variants was obtained from (see Materials and Methods): (i) UniProt database, for pathogenic variants; (ii) a homology-based model, for neutral variants. (PNG 673 kb) [file 12864_2017_3914_MOESM1_ESM.png]

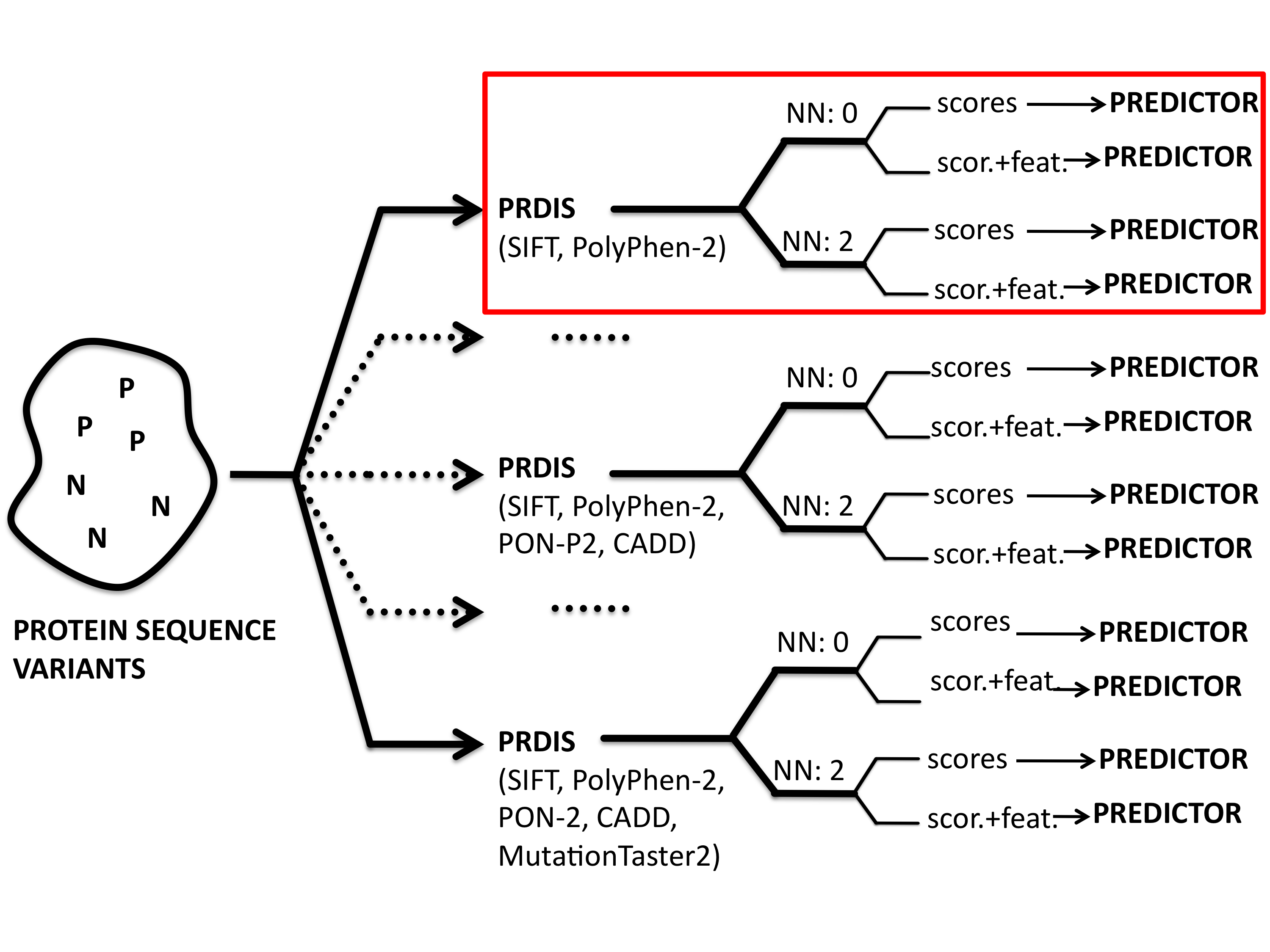

Supplement: Supplementary file 2 — Obtention of specific predictors for PRDIS variants. For each combination of the five reference methods used in this work (SIFT, PolyPhen-2, PON-P2, CADD and MutationTaster2) we obtained PRDIS, the subset of those variants for which the reference predictors disagreed. Then, for each of these PRDIS sets, we produced four different predictors, which differed either in the neural network model or in the neural network input. For the neural network model we tried two options: (i) no hidden layers (NN: 0); and (ii) one hidden with two nodes (NN: 2). For the neural network inputs, we tried two options: (i) the scores of the reference predictors; and (ii) the scores of the reference predictors enriched with three biological features (Blosum62 matrix elements, Shannon’s entropy, Position-specific scoring matrix elements; see Materials and Methods). Boxed in red is the case where PRDIS was obtained using SIFT and PolyPhen-2 as reference methods. (PNG 666 kb) [file 12864_2017_3914_MOESM2_ESM.png]

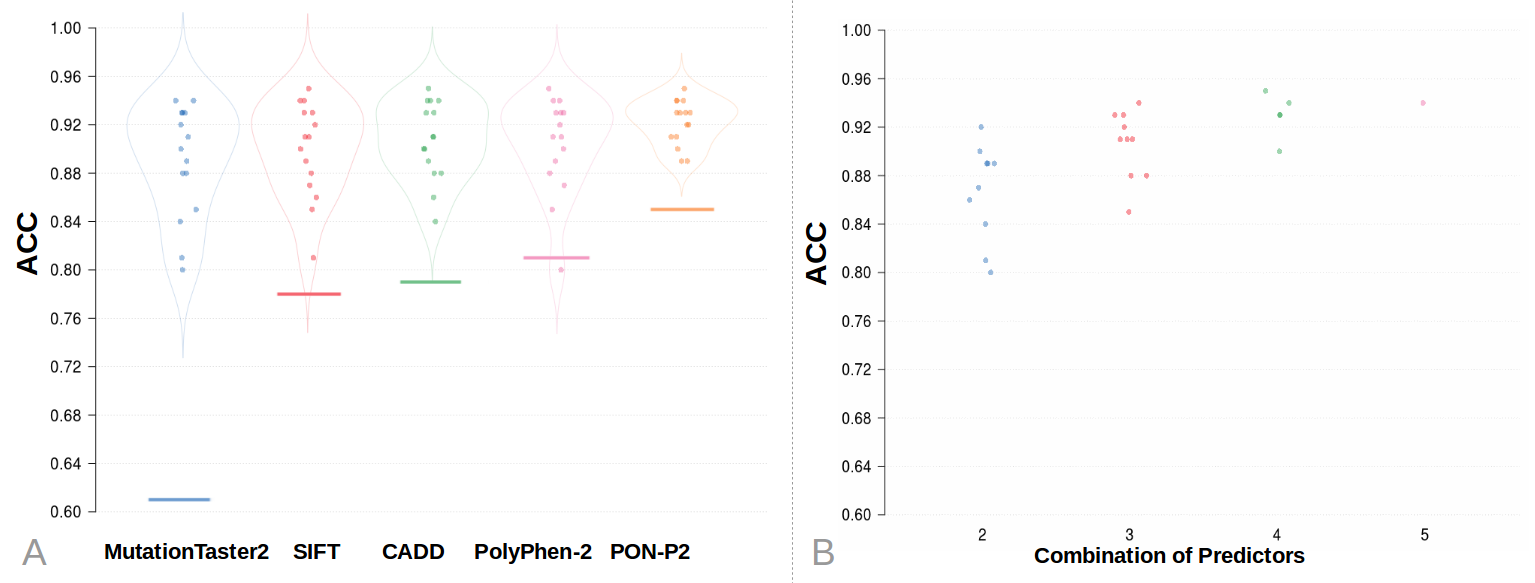

Supplement: Supplementary file 8 — In the coincidence rule (see main text) computational information is accepted as supporting evidence in clinical settings only when the pathogenicity predictions of different methods agree. Here we describe how the success rate of this rule depends on the chosen in silico predictors. (A) Violin plots for the Accuracy grouped by method. Each violin plot corresponds to all possible combinations of reference predictors that include the method shown at the bottom. For example, the first plot to the left represents all combinations of five reference predictors (SIFT, PolyPhen-2, PON-P2, CADD and Mutation Taster2) that include MutationTaster2. (B) Dependence of Accuracy values on the number of predictors used to implement the coincidence rule. (PNG 135 kb) [file 12864_2017_3914_MOESM8_ESM.png]

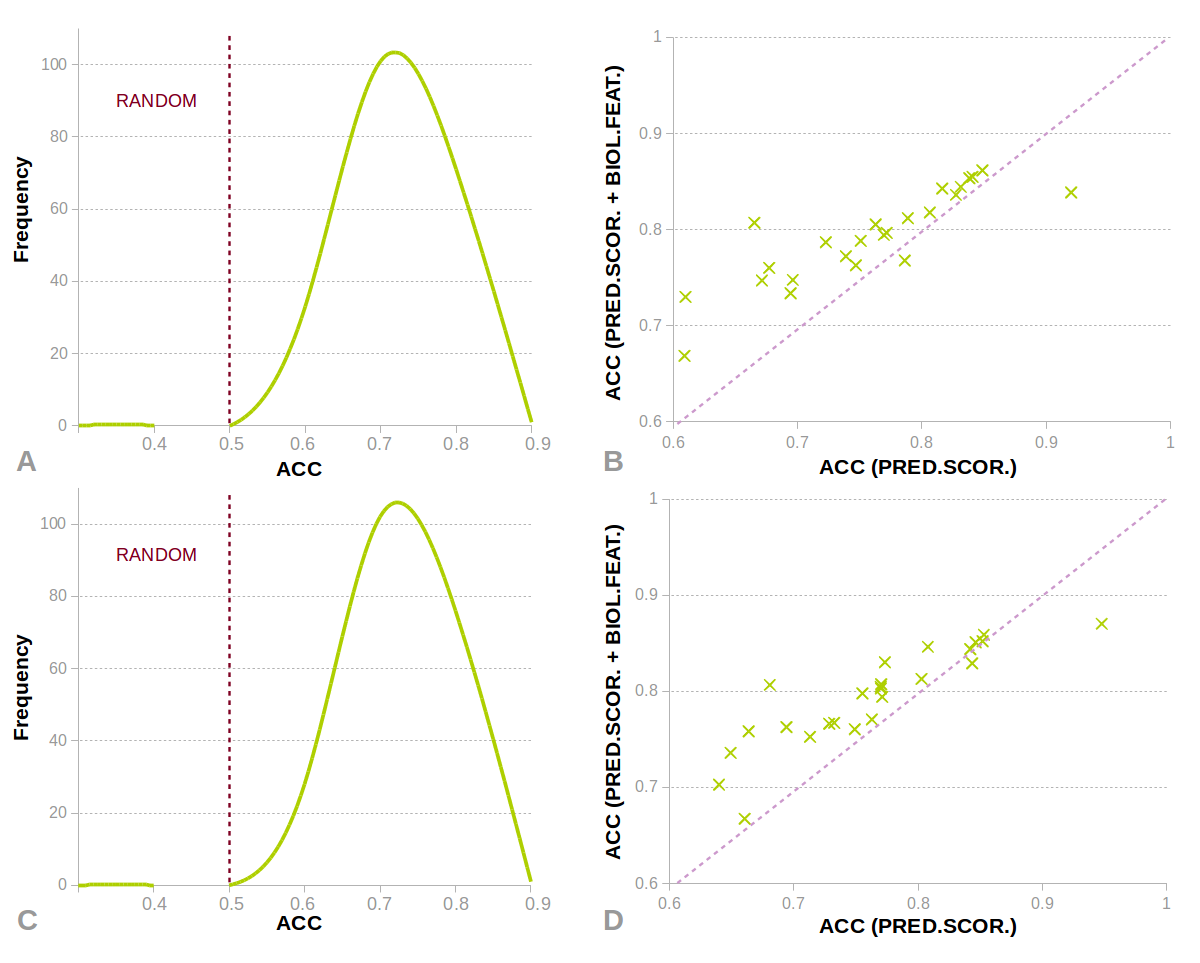

Supplement: Supplementary file 9 — (A) and (C). Frequency distribution of accuracy values for all the specific predictors generated in this work: (A) data for simple neural networks; (C) data for neural networks with one hidden layer and two nodes. Shown with a dashed line is 0.5, the accuracy value for a random predictor. We see that specific predictors are systematically better than the random predictor. (B) and (D). Contribution of the three biochemical/biophysical properties (Blosum62 elements, Shannon’s entropy and Position specific scoring matrix elements; see Materials and Methods) to improve the performance of the specific predictors. Points above the dotted line correspond to cases where use of these properties improves the performance of a specific predictor. We see that this is essentially always the case. (B) and (D) correspond to the simpler and to the one hidden layer neural networks, respectively. (PNG 194 kb) [file 12864_2017_3914_MOESM9_ESM.png]

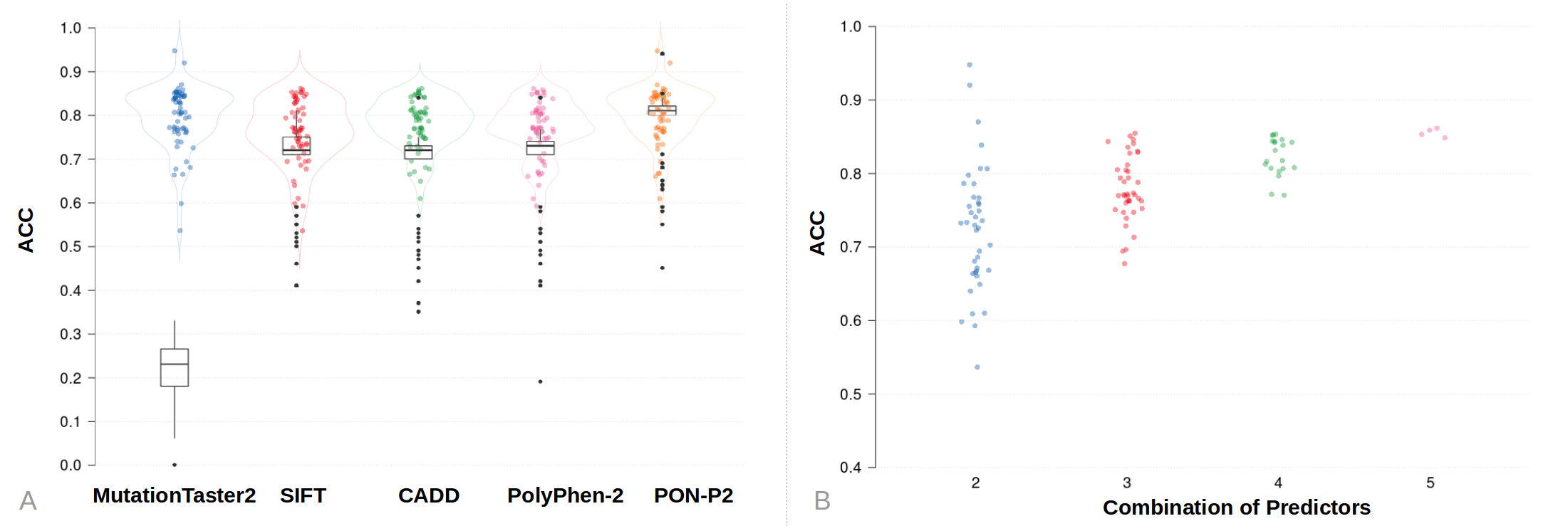

Supplement: Supplementary file 10 — In (A) we compare the performance of PRDIS specific methods, represented with violin plots with that of the reference methods (SIFT, PolyPhen-2, PON-P2, CADD and Mutation Taster2), represented with black boxplots. We see that specific methods are frequently better than reference methods, but there is an increasing overlap between both approaches as the performance of the reference method grows (e.g. in the cases of PON-P2 or PolyPhen-2). (B) Performance depends on the number of reference predictors used: the more predictors are used, the more likely to obtain higher performances. (PNG 219 kb) [file 12864_2017_3914_MOESM10_ESM.png]

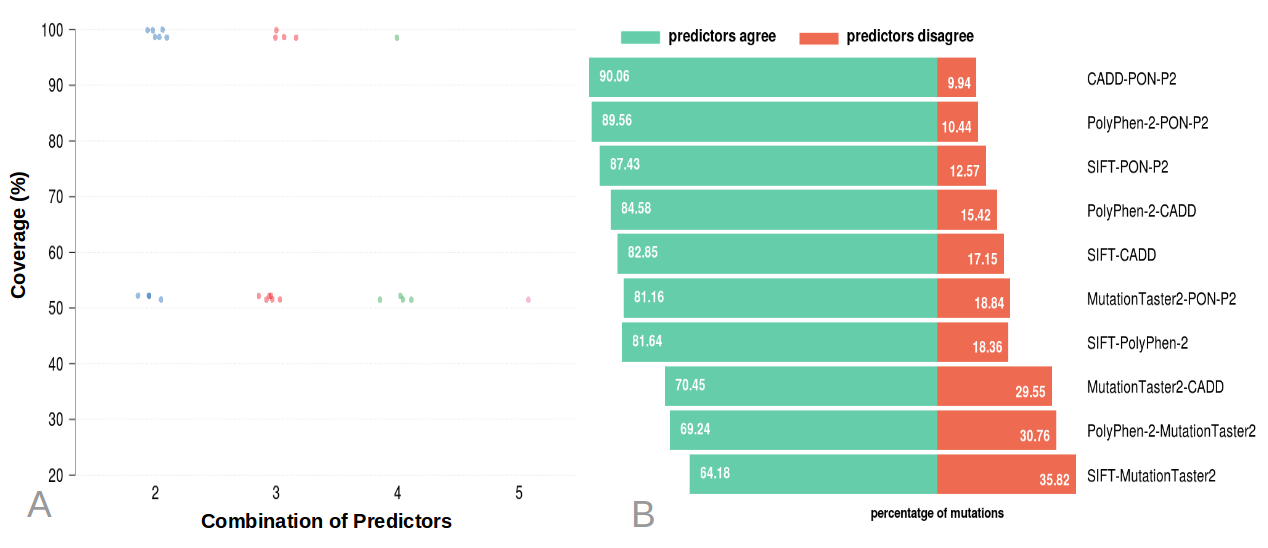

Supplement: Supplementary file 11 — The results in this figure are computed for the subset of amino acid variants resulting from single nucleotide replacements only. (A) Percentage of cases that entered the study. The X-axis corresponds to the number of reference methods combined; each point corresponds to a specific combination of reference predictors (a slight offset is used for clarity purposes). (B) Composition of the PRDIS sets built from the combination of two reference predictors only. Each of the lines (percentage of agreements and disagreements to the left and right, respectively) corresponds to a point in (B), at x = 2. (PNG 115 kb) [file 12864_2017_3914_MOESM11_ESM.png]

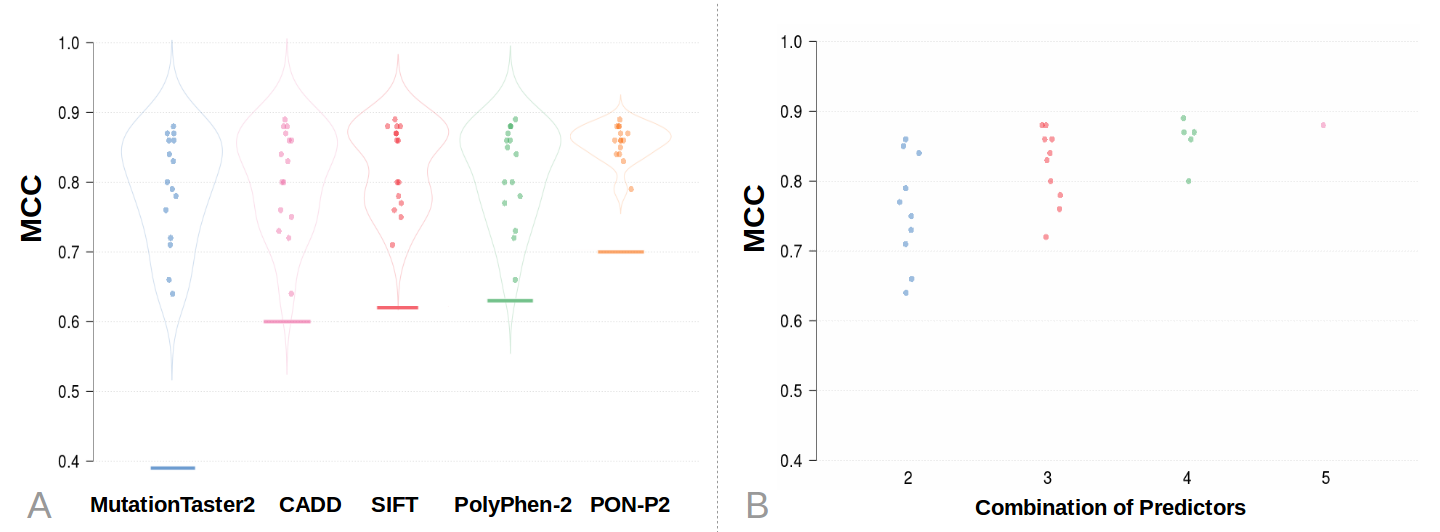

Supplement: Supplementary file 12 — The results in this figure are computed for the subset of amino acid variants resulting from single nucleotide replacements only. In the coincidence rule (see main text) computational information is accepted as supporting evidence in clinical settings only when the pathogenicity predictions of different methods agree. Here we describe how the success rate of this rule depends on the chosen in silico predictors. (A) Violin plots for the Matthews Correlation Coefficients (MCC) grouped by method. Each violin plot corresponds to all possible combinations of reference predictors that include the method shown at the bottom. For example, the first plot to the left represents all combinations of five reference predictors (SIFT, PolyPhen-2, PON-P2, CADD and MutationTaster2) that include MutationTaster2. (B) Dependence of MCC values on the number of predictors used to implement the coincidence rule. (PNG 113 kb) [file 12864_2017_3914_MOESM12_ESM.png]

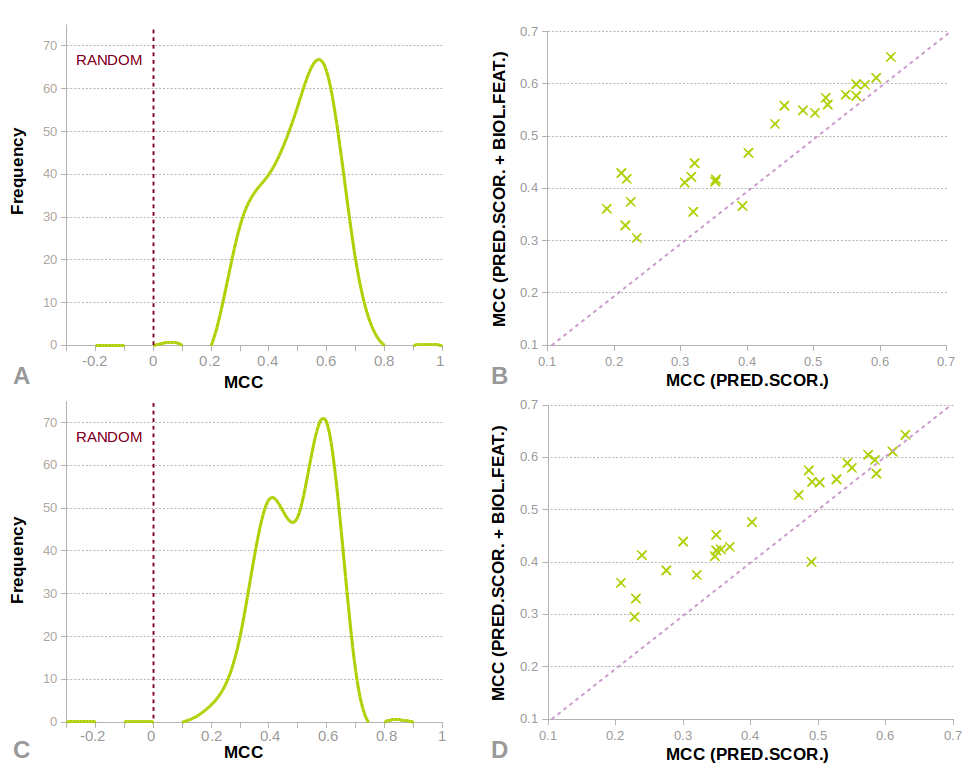

Supplement: Supplementary file 13 — The results in this figure are computed for the subset of amino acid variants resulting from single nucleotide replacements only. (A) and (C). Frequency distribution of MCC values for all the specific predictors generated in this work: (A) data for simple neural networks; (C) data for neural networks with one hidden layer and two nodes. Shown with a dashed line is 0, the MCC value for a random predictor. We see that specific predictors are systematically better than the random predictor. (B) and (D). Contribution of the three biochemical/biophysical properties (Blosum62 elements, Shannon’s entropy and Position specific scoring matrix elements; see Materials and Methods) to improve the performance of the specific predictors. Points above the dotted line correspond to cases where use of these properties improves the performance of a specific predictor. We see that this is essentially always the case. (B) and (D) correspond to the simpler and to the one hidden layer neural networks, respectively. (PNG 172 kb) [file 12864_2017_3914_MOESM13_ESM.png]

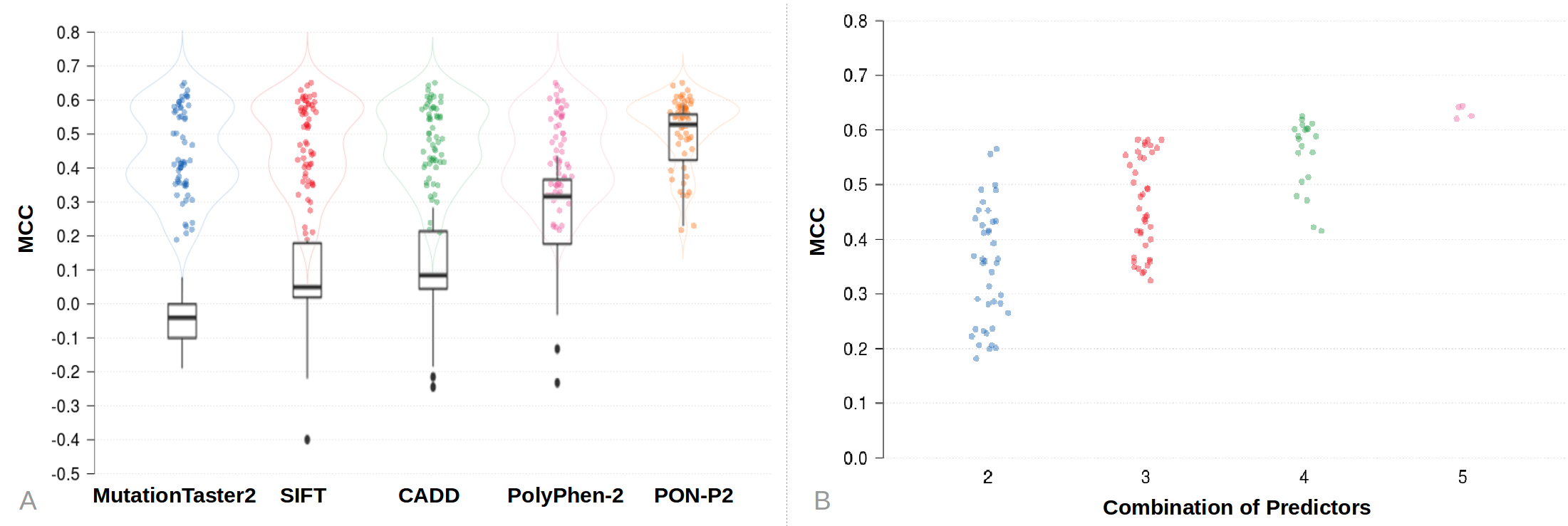

Supplement: Supplementary file 14 — The results in this figure are computed for the subset of amino acid variants resulting from single nucleotide replacements only. In (A) we compare the performance of PRDIS specific methods, represented with violin plots with that of the reference methods (SIFT, PolyPhen-2, PON-P2, CADD and MutationTaster2), represented with black boxplots. We see that specific methods are frequently better than reference methods, but there is an increasing overlap between both approaches as the performance of the reference method grows (e.g. in the cases of PON-P2 or PolyPhen-2). (B) Performance depends on the number of reference predictors combined: the more we use, the more likely we are to obtain higher performances. (PNG 258 kb) [file 12864_2017_3914_MOESM14_ESM.png]

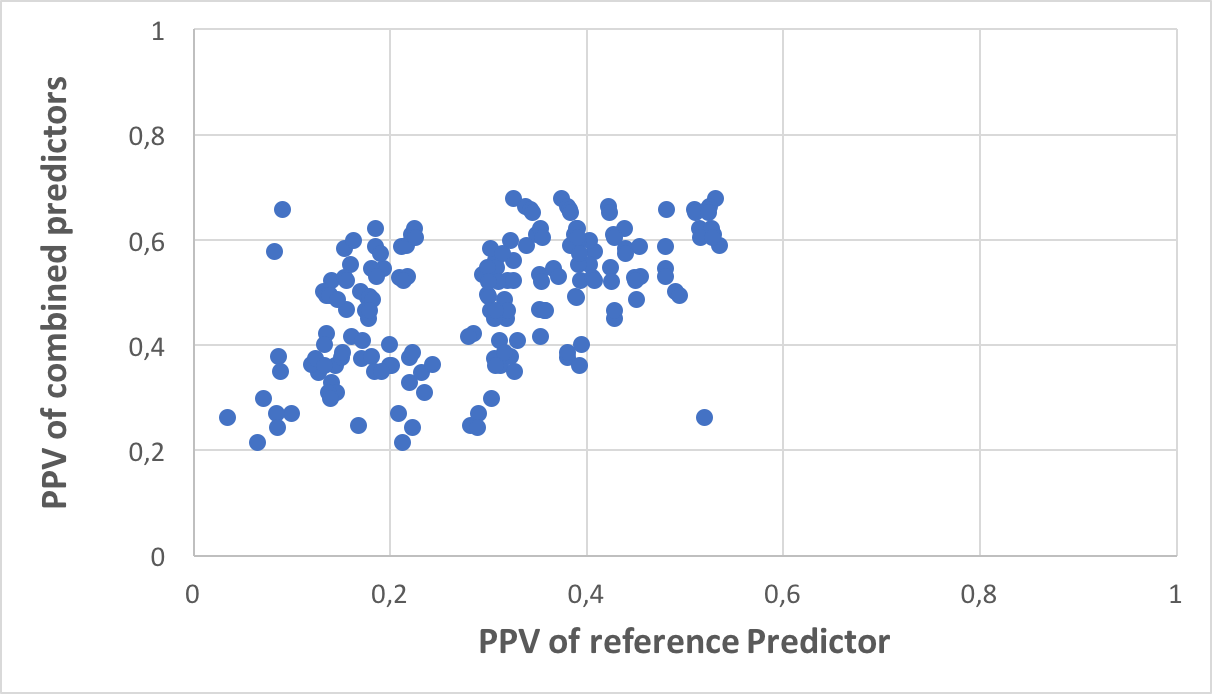

Supplement: Supplementary file 15 — Comparison between PPV values for PRDIS specific and reference predictors. The figure shows that combination of reference methods (specific predictors) gives better PPV than reference methods alone: for only seven cases the reference approach outperformed the specific approach. (PNG 68 kb) [file 12864_2017_3914_MOESM15_ESM.png]

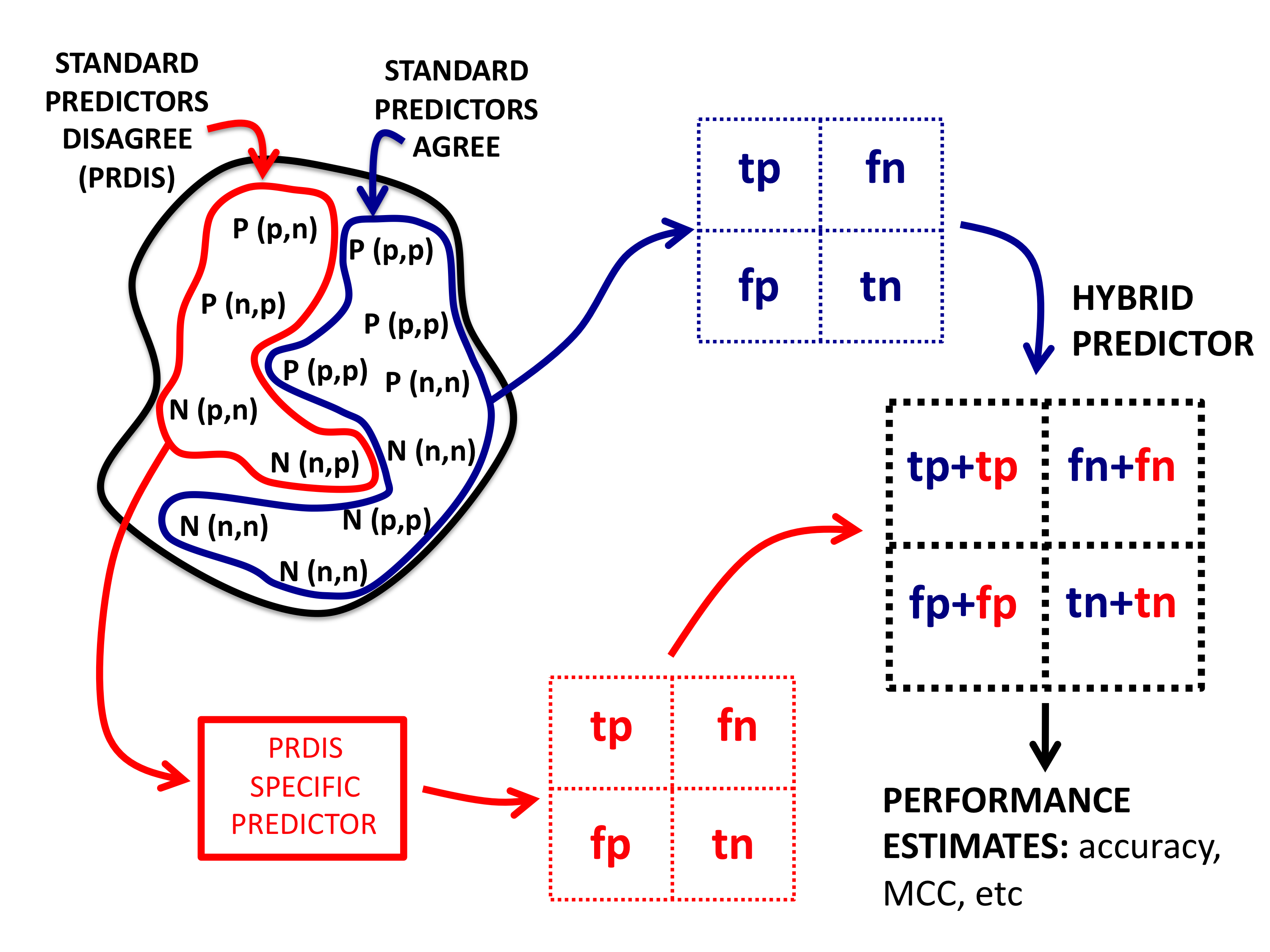

Supplement: Supplementary file 16 — A hybrid predictor. A hybrid method is implicitly defined if the coincidence rule is used as a pre-classification step. In this method, the variants for which standard methods agree will be assigned this coinciding prediction; for PRDIS variants, a prediction will be obtained from the PRDIS specific method. The final performance of this hybrid method is obtained by combining that of the two cases. (PNG 607 kb) [file 12864_2017_3914_MOESM16_ESM.png]
