# Supplementary material for: Development of pathogenicity predictors specific for variants that do not comply with clinical guidelines for the use of computational evidence
Source: BMC Genomics. 2017 Aug 11;18(Suppl 5):569. doi: 10.1186/s12864-017-3914-0 (PMC5558188; doi:10.1186/s12864-017-3914-0)
Supplement: Supplementary file 5 — Success rate of the coincidence rule, for the all the different combinations of reference predictors (SIFT, PolyPhen-2, PON-P2, CADD and MutationTaster2). The performance measures are the six standard measures (MCC, accuracy, sensitivity, specificity, PPV and NPV) described in the Materials and Methods section. We give: the raw TP, TN, FP and FN values; the coverage relative to the original dataset of 59,442 variants (VS228) and the number of cases where the predictors coincide. (PDF 28 kb) [file 12864_2017_3914_MOESM5_ESM.pdf]

| Combination Of Predictors | | | | | | Cases with predictions for all methods (coverage) | | Cases with coinciding predictions | | | | | | | | | | | |
|---|---|---|---|---|---|---|---|---|---|---|---|---|---|---|---|---|---|---|---|
| | | | | | | Number of cases | Percentage (relative to 59442) | Number of cases | Percentage | TP | FP | TN | FN | SENS | SPEC | ACC | MCC | PPV | NPV |
| **SIFT** | | | | | | 57700 | 97.07 | 57700 | 100.00 | 12294 | 8897 | 33430 | 3079 | 0.800 | 0.790 | 0.792 | 0.541 | 0.580 | 0.916 |
| **PolyPhen-2_HDIV** | | | | | | 59089 | 99.41 | 59089 | 100.00 | 14088 | 11343 | 32198 | 1460 | 0.906 | 0.739 | 0.783 | 0.574 | 0.554 | 0.957 |
| **PolyPhen-2_HVAR** | | | | | | 59089 | 99.41 | 59089 | 100.00 | 13536 | 8506 | 35035 | 2012 | 0.871 | 0.805 | 0.822 | 0.615 | 0.614 | 0.946 |
| **MutationTaster2** | | | | | | 32765 | 55.12 | 32765 | 100.00 | 8999 | 12236 | 10951 | 579 | 0.940 | 0.472 | 0.609 | 0.392 | 0.424 | 0.950 |
| **CADD** | | | | | | 32765 | 55.12 | 32765 | 100.00 | 8047 | 4618 | 18569 | 1531 | 0.840 | 0.801 | 0.812 | 0.599 | 0.635 | 0.924 |
| **PON-P2** | | | | | | 30799 | 51.81 | 30799 | 100.00 | 9091 | 4107 | 17051 | 550 | 0.943 | 0.806 | 0.849 | 0.702 | 0.689 | 0.969 |
| **SIFT** | **PolyPhen-2_HDIV** | | | | | 57349 | 96.48 | 46213 | 80.58 | 11647 | 5648 | 27984 | 934 | 0.926 | 0.832 | 0.858 | 0.697 | 0.673 | 0.968 |
| **SIFT** | **PolyPhen-2_HVAR** | | | | | 57349 | 96.48 | 47465 | 82.77 | 11405 | 4857 | 29971 | 1232 | 0.903 | 0.861 | 0.872 | 0.711 | 0.701 | 0.961 |
| **SIFT** | **MutationTaster2** | | | | | 32741 | 55.08 | 20990 | 64.11 | 7483 | 2945 | 10192 | 370 | 0.953 | 0.776 | 0.842 | 0.705 | 0.718 | 0.965 |
| **SIFT** | **CADD** | | | | | 32741 | 55.08 | 27109 | 82.80 | 6985 | 2219 | 17081 | 824 | 0.894 | 0.885 | 0.888 | 0.745 | 0.759 | 0.954 |
| **SIFT** | **PON-P2** | | | | | 29718 | 49.99 | 25402 | 85.48 | 7836 | 2287 | 15002 | 277 | 0.966 | 0.868 | 0.899 | 0.794 | 0.774 | 0.982 |
| **PolyPhen-2_HDIV** | **PolyPhen-2_HVAR** | | | | | 59089 | 99.41 | 55332 | 93.64 | 13484 | 8374 | 32066 | 1408 | 0.905 | 0.793 | 0.823 | 0.634 | 0.617 | 0.958 |
| **PolyPhen-2_HDIV** | **MutationTaster2** | | | | | 32753 | 55.10 | 22673 | 69.22 | 8433 | 4104 | 9852 | 284 | 0.967 | 0.706 | 0.806 | 0.659 | 0.673 | 0.972 |
| **PolyPhen-2_HDIV** | **CADD** | | | | | 32753 | 55.10 | 27697 | 84.56 | 7784 | 2983 | 16343 | 587 | 0.930 | 0.846 | 0.871 | 0.731 | 0.723 | 0.965 |
| **PolyPhen-2_HDIV** | **PON-P2** | | | | | 30651 | 51.56 | 27118 | 88.47 | 8667 | 2866 | 15372 | 213 | 0.976 | 0.843 | 0.886 | 0.777 | 0.751 | 0.986 |
| **PolyPhen-2_HVAR** | **MutationTaster2** | | | | | 32753 | 55.10 | 22138 | 67.59 | 8162 | 3242 | 10386 | 348 | 0.959 | 0.762 | 0.838 | 0.702 | 0.716 | 0.968 |
| **PolyPhen-2_HVAR** | **CADD** | | | | | 32753 | 55.10 | 28210 | 86.13 | 7598 | 2560 | 17316 | 736 | 0.912 | 0.871 | 0.883 | 0.744 | 0.748 | 0.959 |
| **PolyPhen-2_HVAR** | **PON-P2** | | | | | 30651 | 51.56 | 27460 | 89.59 | 8526 | 2556 | 16112 | 266 | 0.970 | 0.863 | 0.897 | 0.792 | 0.769 | 0.984 |
| **MutationTaster2** | **CADD** | | | | | 32765 | 55.12 | 23083 | 70.45 | 7912 | 4197 | 10530 | 444 | 0.947 | 0.715 | 0.799 | 0.637 | 0.653 | 0.960 |
| **MutationTaster2** | **PON-P2** | | | | | 17118 | 28.80 | 13893 | 81.16 | 5487 | 1470 | 6817 | 119 | 0.979 | 0.823 | 0.886 | 0.786 | 0.789 | 0.983 |
| **CADD** | **PON-P2** | | | | | 17118 | 28.80 | 15417 | 90.06 | 5116 | 1041 | 9080 | 180 | 0.966 | 0.897 | 0.921 | 0.837 | 0.831 | 0.981 |
| **SIFT** | **PolyPhen-2_HDIV** | **PolyPhen-2_HVAR** | | | | 57349 | 96.48 | 45006 | 78.48 | 11366 | 4794 | 27923 | 923 | 0.925 | 0.853 | 0.873 | 0.723 | 0.703 | 0.968 |
| **SIFT** | **PolyPhen-2_HDIV** | **MutationTaster2** | | | | 32729 | 55.06 | 18817 | 57.49 | 7267 | 1982 | 9340 | 228 | 0.970 | 0.825 | 0.883 | 0.778 | 0.786 | 0.976 |
| **SIFT** | **PolyPhen-2_HDIV** | **CADD** | | | | 32729 | 55.06 | 24385 | 74.51 | 6839 | 1719 | 15366 | 461 | 0.937 | 0.899 | 0.911 | 0.802 | 0.799 | 0.971 |
| **SIFT** | **PolyPhen-2_HDIV** | **PON-P2** | | | | 29572 | 49.75 | 23554 | 79.65 | 7574 | 1945 | 13859 | 176 | 0.977 | 0.877 | 0.910 | 0.818 | 0.796 | 0.987 |
| **SIFT** | **PolyPhen-2_HVAR** | **MutationTaster2** | | | | 32729 | 55.06 | 18993 | 58.03 | 7146 | 1787 | 9790 | 270 | 0.964 | 0.846 | 0.892 | 0.791 | 0.800 | 0.973 |
| **SIFT** | **PolyPhen-2_HVAR** | **CADD** | | | | 32729 | 55.06 | 25084 | 76.64 | 6745 | 1577 | 16201 | 561 | 0.923 | 0.911 | 0.915 | 0.805 | 0.811 | 0.967 |
| **SIFT** | **PolyPhen-2_HVAR** | **PON-P2** | | | | 29572 | 49.75 | 23961 | 81.03 | 7502 | 1822 | 14428 | 209 | 0.973 | 0.888 | 0.915 | 0.825 | 0.805 | 0.986 |
| **SIFT** | **MutationTaster2** | **CADD** | | | | 32741 | 55.08 | 19210 | 58.67 | 6899 | 2074 | 9916 | 321 | 0.956 | 0.827 | 0.875 | 0.760 | 0.769 | 0.969 |
| **SIFT** | **MutationTaster2** | **PON-P2** | | | | 17095 | 28.76 | 12270 | 71.78 | 4874 | 801 | 6497 | 98 | 0.980 | 0.890 | 0.927 | 0.857 | 0.859 | 0.985 |
| **SIFT** | **CADD** | **PON-P2** | | | | 17095 | 28.76 | 14113 | 82.56 | 4620 | 683 | 8671 | 139 | 0.971 | 0.927 | 0.942 | 0.876 | 0.871 | 0.984 |
| **PolyPhen-2_HDIV** | **PolyPhen-2_HVAR** | **MutationTaster2** | | | | 32753 | 55.10 | 21459 | 65.52 | 8145 | 3198 | 9836 | 280 | 0.967 | 0.755 | 0.838 | 0.706 | 0.718 | 0.972 |
| **PolyPhen-2_HDIV** | **PolyPhen-2_HVAR** | **CADD** | | | | 32753 | 55.10 | 27007 | 82.46 | 7583 | 2533 | 16310 | 581 | 0.929 | 0.866 | 0.885 | 0.754 | 0.750 | 0.966 |
| **PolyPhen-2_HDIV** | **PolyPhen-2_HVAR** | **PON-P2** | | | | 30651 | 51.56 | 26594 | 86.76 | 8509 | 2526 | 15346 | 213 | 0.976 | 0.859 | 0.897 | 0.795 | 0.771 | 0.986 |
| **PolyPhen-2_HDIV** | **MutationTaster2** | **CADD** | | | | 32753 | 55.10 | 20347 | 62.12 | 7669 | 2793 | 9621 | 264 | 0.967 | 0.775 | 0.850 | 0.724 | 0.733 | 0.973 |
| **PolyPhen-2_HDIV** | **MutationTaster2** | **PON-P2** | | | | 17116 | 28.79 | 12808 | 74.83 | 5339 | 997 | 6387 | 85 | 0.984 | 0.865 | 0.916 | 0.839 | 0.843 | 0.987 |
| **PolyPhen-2_HDIV** | **CADD** | **PON-P2** | | | | 17116 | 28.79 | 14445 | 84.39 | 5034 | 843 | 8453 | 115 | 0.978 | 0.909 | 0.934 | 0.865 | 0.857 | 0.987 |
| **PolyPhen-2_HVAR** | **MutationTaster2** | **CADD** | | | | 32753 | 55.10 | 20336 | 62.09 | 7493 | 2426 | 10099 | 318 | 0.959 | 0.806 | 0.865 | 0.745 | 0.755 | 0.969 |
| **PolyPhen-2_HVAR** | **MutationTaster2** | **PON-P2** | | | | 17116 | 28.79 | 12887 | 75.29 | 5264 | 892 | 6634 | 97 | 0.982 | 0.881 | 0.923 | 0.852 | 0.855 | 0.986 |
| **PolyPhen-2_HVAR** | **CADD** | **PON-P2** | | | | 17116 | 28.79 | 14672 | 85.72 | 4979 | 772 | 8788 | 133 | 0.974 | 0.919 | 0.938 | 0.872 | 0.866 | 0.985 |
| **MutationTaster2** | **CADD** | **PON-P2** | | | | 17118 | 28.80 | 12874 | 75.21 | 5079 | 1027 | 6665 | 103 | 0.980 | 0.866 | 0.912 | 0.831 | 0.832 | 0.985 |
| **SIFT** | **PolyPhen-2_HDIV** | **PolyPhen-2_HVAR** | **MutationTaster2** | | | 32729 | 55.06 | 18453 | 56.38 | 7132 | 1764 | 9330 | 227 | 0.969 | 0.841 | 0.892 | 0.794 | 0.802 | 0.976 |
| **SIFT** | **PolyPhen-2_HDIV** | **PolyPhen-2_HVAR** | **CADD** | | | 32729 | 55.06 | 24100 | 73.64 | 6731 | 1563 | 15348 | 458 | 0.936 | 0.908 | 0.916 | 0.813 | 0.812 | 0.971 |
| **SIFT** | **PolyPhen-2_HDIV** | **PolyPhen-2_HVAR** | **PON-P2** | | | 29572 | 49.75 | 23313 | 78.83 | 7490 | 1805 | 13842 | 176 | 0.977 | 0.885 | 0.915 | 0.827 | 0.806 | 0.987 |
| **SIFT** | **PolyPhen-2_HDIV** | **MutationTaster2** | **CADD** | | | 32729 | 55.06 | 17796 | 54.37 | 6760 | 1643 | 9178 | 215 | 0.969 | 0.848 | 0.896 | 0.799 | 0.804 | 0.977 |
| **SIFT** | **PolyPhen-2_HDIV** | **MutationTaster2** | **PON-P2** | | | 17093 | 28.76 | 11688 | 68.38 | 4794 | 681 | 6137 | 76 | 0.984 | 0.900 | 0.935 | 0.874 | 0.876 | 0.988 |
| **SIFT** | **PolyPhen-2_HDIV** | **CADD** | **PON-P2** | | | 17093 | 28.76 | 13410 | 78.45 | 4564 | 612 | 8128 | 106 | 0.977 | 0.930 | 0.946 | 0.888 | 0.882 | 0.987 |
| **SIFT** | **PolyPhen-2_HVAR** | **MutationTaster2** | **CADD** | | | 32729 | 55.06 | 18026 | 55.08 | 6672 | 1515 | 9586 | 253 | 0.963 | 0.864 | 0.902 | 0.808 | 0.815 | 0.974 |
| **SIFT** | **PolyPhen-2_HVAR** | **MutationTaster2** | **PON-P2** | | | 17093 | 28.76 | 11846 | 69.30 | 4759 | 648 | 6354 | 85 | 0.982 | 0.907 | 0.938 | 0.878 | 0.880 | 0.987 |
| **SIFT** | **PolyPhen-2_HVAR** | **CADD** | **PON-P2** | | | 17093 | 28.76 | 13663 | 79.93 | 4536 | 580 | 8427 | 120 | 0.974 | 0.936 | 0.949 | 0.891 | 0.887 | 0.986 |
| **SIFT** | **MutationTaster2** | **CADD** | **PON-P2** | | | 17095 | 28.76 | 11747 | 68.72 | 4594 | 675 | 6389 | 89 | 0.981 | 0.904 | 0.935 | 0.872 | 0.872 | 0.986 |
| **PolyPhen-2_HDIV** | **PolyPhen-2_HVAR** | **MutationTaster2** | **CADD** | | | 32753 | 55.10 | 19746 | 60.29 | 7480 | 2399 | 9605 | 262 | 0.966 | 0.800 | 0.865 | 0.748 | 0.757 | 0.973 |
| **PolyPhen-2_HDIV** | **PolyPhen-2_HVAR** | **MutationTaster2** | **PON-P2** | | | 17116 | 28.79 | 12602 | 73.63 | 5254 | 882 | 6381 | 85 | 0.984 | 0.879 | 0.923 | 0.853 | 0.856 | 0.987 |
| **PolyPhen-2_HDIV** | **PolyPhen-2_HVAR** | **CADD** | **PON-P2** | | | 17116 | 28.79 | 14296 | 83.52 | 4971 | 765 | 8445 | 115 | 0.977 | 0.917 | 0.938 | 0.874 | 0.867 | 0.987 |
| **PolyPhen-2_HDIV** | **MutationTaster2** | **CADD** | **PON-P2** | | | 17116 | 28.79 | 12196 | 71.25 | 5000 | 831 | 6285 | 80 | 0.984 | 0.883 | 0.925 | 0.856 | 0.857 | 0.987 |
| **PolyPhen-2_HVAR** | **MutationTaster2** | **CADD** | **PON-P2** | | | 17116 | 28.79 | 12312 | 71.93 | 4946 | 762 | 6513 | 91 | 0.982 | 0.895 | 0.931 | 0.865 | 0.867 | 0.986 |
| **SIFT** | **PolyPhen-2_HDIV** | **PolyPhen-2_HVAR** | **MutationTaster2** | **CADD** | | 32729 | 55.06 | 17543 | 53.60 | 6660 | 1501 | 9168 | 214 | 0.969 | 0.859 | 0.902 | 0.811 | 0.816 | 0.977 |
| **SIFT** | **PolyPhen-2_HDIV** | **PolyPhen-2_HVAR** | **MutationTaster2** | **PON-P2** | | 17093 | 28.76 | 11602 | 67.88 | 4751 | 642 | 6133 | 76 | 0.984 | 0.905 | 0.938 | 0.879 | 0.881 | 0.988 |
| **SIFT** | **PolyPhen-2_HDIV** | **PolyPhen-2_HVAR** | **CADD** | **PON-P2** | | 17093 | 28.76 | 13334 | 78.01 | 4529 | 576 | 8123 | 106 | 0.977 | 0.934 | 0.949 | 0.892 | 0.887 | 0.987 |
| **SIFT** | **PolyPhen-2_HDIV** | **MutationTaster2** | **CADD** | **PON-P2** | | 17093 | 28.76 | 11278 | 65.98 | 4540 | 604 | 6062 | 72 | 0.984 | 0.909 | 0.940 | 0.882 | 0.883 | 0.988 |
| **SIFT** | **PolyPhen-2_HVAR** | **MutationTaster2** | **CADD** | **PON-P2** | | 17093 | 28.76 | 11432 | 66.88 | 4513 | 574 | 6265 | 80 | 0.983 | 0.916 | 0.943 | 0.887 | 0.887 | 0.987 |
| **PolyPhen-2_HDIV** | **PolyPhen-2_HVAR** | **MutationTaster2** | **CADD** | **PON-P2** | | 17116 | 28.79 | 12052 | 70.41 | 4938 | 755 | 6279 | 80 | 0.984 | 0.893 | 0.931 | 0.866 | 0.867 | 0.987 |
| **SIFT** | **PolyPhen-2_HDIV** | **PolyPhen-2_HVAR** | **MutationTaster2** | **CADD** | **PON-P2** | 17093 | 28.76 | 11206 | 65.56 | 4506 | 570 | 6058 | 72 | 0.984 | 0.914 | 0.943 | 0.887 | 0.888 | 0.988 |
