# Supplementary material for: Development of pathogenicity predictors specific for variants that do not comply with clinical guidelines for the use of computational evidence
Source: BMC Genomics. 2017 Aug 11;18(Suppl 5):569. doi: 10.1186/s12864-017-3914-0 (PMC5558188; doi:10.1186/s12864-017-3914-0)
Supplement: Supplementary file 6 — Prediction performance for the PRDIS specific predictors in this work for VS228; each corresponds to a different combination of the reference predictors (SIFT, PolyPhen-2, PON-P2, CADD and MutationTaster2). The performance measures are the six standard measures (MCC, accuracy, sensitivity, specificity, PPV and NPV) described in the Materials and Methods section. We also give: the total number and the percentage of cases, and the raw TP, TN, FP and FN values. (PDF 26 kb) [file 12864_2017_3914_MOESM6_ESM.pdf]

PRDIS SPECIFIC PREDICTORS						Number of cases											
						Total	%	TP	FP	TN	FN	SENS	SPEC	ACC	MCC	PPV	NPV
**SIFT**	**PolyPhen-2_HDIV**					11136	19.42	1895	2589	5930	722	0.724	0.696	0.703	0.363	0.423	0.891
**SIFT**	**PolyPhen-2_HVAR**					9884	17.23	1808	2049	5284	748	0.707	0.721	0.717	0.384	0.469	0.876
**SIFT**	**MutationTaster2**					11751	35.89	1279	2606	7423	443	0.743	0.740	0.741	0.363	0.329	0.944
**SIFT**	**CADD**					5632	17.20	1269	992	2874	497	0.719	0.743	0.736	0.437	0.561	0.853
**SIFT**	**PON-P2**					4316	14.52	894	650	2239	301	0.748	0.775	0.767	0.491	0.579	0.881
**PolyPhen-2_HDIV**	**PolyPhen-2_HVAR**					3757	6.36	456	1375	1706	186	0.710	0.554	0.581	0.199	0.249	0.902
**PolyPhen-2_HDIV**	**MutationTaster2**					10080	30.78	602	2181	7041	256	0.702	0.764	0.758	0.290	0.216	0.965
**PolyPhen-2_HDIV**	**CADD**					5056	15.44	840	847	3005	364	0.698	0.780	0.760	0.432	0.498	0.892
**PolyPhen-2_HDIV**	**PON-P2**					3533	11.53	353	950	1812	130	0.731	0.656	0.667	0.281	0.271	0.933
**PolyPhen-2_HVAR**	**MutationTaster2**					10615	32.41	719	1937	7605	334	0.683	0.797	0.786	0.331	0.271	0.958
**PolyPhen-2_HVAR**	**CADD**					4543	13.87	923	955	2351	314	0.746	0.711	0.721	0.413	0.491	0.882
**PolyPhen-2_HVAR**	**PON-P2**					3191	10.41	391	728	1628	180	0.685	0.691	0.690	0.306	0.349	0.900
**MutationTaster2**	**CADD**					9682	29.55	877	1530	6930	345	0.718	0.819	0.806	0.412	0.364	0.953
**MutationTaster2**	**PON-P2**					3225	18.84	122	340	2551	58	0.678	0.882	0.870	0.368	0.264	0.978
**CADD**	**PON-P2**					1701	9.94	394	205	847	110	0.782	0.805	0.798	0.564	0.658	0.885
**SIFT**	**PolyPhen-2_HDIV**	**PolyPhen-2_HVAR**				12343	21.52	2065	2881	6549	834	0.712	0.694	0.699	0.352	0.418	0.887
**SIFT**	**PolyPhen-2_HDIV**	**MutationTaster2**				13912	42.51	1613	2839	8996	464	0.777	0.760	0.763	0.410	0.362	0.951
**SIFT**	**PolyPhen-2_HDIV**	**CADD**				8344	25.49	1605	1397	4675	667	0.706	0.770	0.753	0.442	0.535	0.875
**SIFT**	**PolyPhen-2_HDIV**	**PON-P2**				6018	20.35	1115	1011	3315	307	0.784	0.766	0.771	0.492	0.524	0.915
**SIFT**	**PolyPhen-2_HVAR**	**MutationTaster2**				13736	41.97	1661	2732	8849	486	0.774	0.764	0.766	0.419	0.378	0.948
**SIFT**	**PolyPhen-2_HVAR**	**CADD**				7645	23.36	1642	1347	4039	619	0.726	0.750	0.743	0.445	0.549	0.867
**SIFT**	**PolyPhen-2_HVAR**	**PON-P2**				5611	18.97	1134	909	2986	324	0.778	0.767	0.770	0.499	0.555	0.902
**SIFT**	**MutationTaster2**	**CADD**				13531	41.33	1826	2636	8540	529	0.775	0.764	0.766	0.435	0.409	0.942
**SIFT**	**MutationTaster2**	**PON-P2**				4825	28.22	689	680	3138	111	0.861	0.822	0.829	0.566	0.503	0.966
**SIFT**	**CADD**	**PON-P2**				2982	17.44	830	392	1384	182	0.820	0.779	0.794	0.581	0.679	0.884
**PolyPhen-2_HDIV**	**PolyPhen-2_HVAR**	**MutationTaster2**				11294	34.48	830	2559	7571	307	0.730	0.747	0.746	0.314	0.245	0.961
**PolyPhen-2_HDIV**	**PolyPhen-2_HVAR**	**CADD**				5746	17.54	951	976	3351	451	0.678	0.774	0.751	0.412	0.494	0.881
**PolyPhen-2_HDIV**	**PolyPhen-2_HVAR**	**PON-P2**				4057	13.24	455	1008	2111	180	0.717	0.677	0.684	0.302	0.311	0.921
**PolyPhen-2_HDIV**	**MutationTaster2**	**CADD**				12406	37.88	1203	2009	8755	439	0.733	0.813	0.803	0.422	0.375	0.952
**PolyPhen-2_HDIV**	**MutationTaster2**	**PON-P2**				4308	25.17	260	606	3135	89	0.745	0.838	0.830	0.399	0.300	0.972
**PolyPhen-2_HDIV**	**CADD**	**PON-P2**				2671	15.61	489	349	1492	132	0.787	0.810	0.805	0.548	0.584	0.919
**PolyPhen-2_HVAR**	**MutationTaster2**	**CADD**				12417	37.91	1290	1922	8729	466	0.735	0.820	0.808	0.441	0.402	0.949
**PolyPhen-2_HVAR**	**MutationTaster2**	**PON-P2**				4229	24.71	304	499	3111	103	0.747	0.862	0.850	0.459	0.379	0.968
**PolyPhen-2_HVAR**	**CADD**	**PON-P2**				2444	14.28	498	302	1288	156	0.761	0.810	0.796	0.542	0.623	0.892
**MutationTaster2**	**CADD**	**PON-P2**				4244	24.79	507	517	2942	88	0.852	0.851	0.851	0.572	0.495	0.971
**SIFT**	**PolyPhen-2_HDIV**	**PolyPhen-2_HVAR**	**MutationTaster2**			14276	43.62	1656	2621	9438	547	0.752	0.783	0.778	0.421	0.387	0.945
**SIFT**	**PolyPhen-2_HDIV**	**PolyPhen-2_HVAR**	**CADD**			8629	26.36	1752	1604	4639	623	0.738	0.743	0.742	0.441	0.522	0.882
**SIFT**	**PolyPhen-2_HDIV**	**PolyPhen-2_HVAR**	**PON-P2**			6259	21.17	1060	707	3774	441	0.706	0.842	0.808	0.521	0.600	0.895
**SIFT**	**PolyPhen-2_HDIV**	**MutationTaster2**	**CADD**			14933	45.63	1936	2215	10121	661	0.745	0.820	0.807	0.479	0.466	0.939
**SIFT**	**PolyPhen-2_HDIV**	**MutationTaster2**	**PON-P2**			5405	31.62	766	638	3639	127	0.858	0.851	0.852	0.602	0.546	0.966
**SIFT**	**PolyPhen-2_HDIV**	**CADD**	**PON-P2**			3683	21.55	902	457	1911	191	0.825	0.807	0.813	0.602	0.664	0.909
**SIFT**	**PolyPhen-2_HVAR**	**MutationTaster2**	**CADD**			14703	44.92	2051	2484	9575	589	0.777	0.794	0.791	0.474	0.452	0.942
**SIFT**	**PolyPhen-2_HVAR**	**MutationTaster2**	**PON-P2**			5247	30.70	758	531	3573	157	0.828	0.871	0.863	0.618	0.588	0.958
**SIFT**	**PolyPhen-2_HVAR**	**CADD**	**PON-P2**			3430	20.07	943	487	1625	161	0.854	0.769	0.799	0.596	0.659	0.910
**SIFT**	**MutationTaster2**	**CADD**	**PON-P2**			5348	31.28	935	651	3398	151	0.861	0.839	0.844	0.619	0.590	0.957
**PolyPhen-2_HDIV**	**PolyPhen-2_HVAR**	**MutationTaster2**	**CADD**			13007	39.71	1393	2453	8714	431	0.764	0.780	0.778	0.414	0.362	0.953
**PolyPhen-2_HDIV**	**PolyPhen-2_HVAR**	**MutationTaster2**	**PON-P2**			4514	26.37	328	606	3250	100	0.766	0.843	0.835	0.442	0.351	0.970
**PolyPhen-2_HDIV**	**PolyPhen-2_HVAR**	**CADD**	**PON-P2**			2820	16.48	539	396	1527	140	0.794	0.794	0.794	0.538	0.576	0.916
**PolyPhen-2_HDIV**	**MutationTaster2**	**CADD**	**PON-P2**			4920	28.75	579	611	3394	111	0.839	0.847	0.846	0.559	0.487	0.968
**PolyPhen-2_HVAR**	**MutationTaster2**	**CADD**	**PON-P2**			4804	28.07	601	532	3326	129	0.823	0.862	0.856	0.581	0.530	0.963
**SIFT**	**PolyPhen-2_HDIV**	**PolyPhen-2_HVAR**	**MutationTaster2**	**CADD**		15186	46.40	2013	2303	10183	677	0.748	0.816	0.804	0.477	0.466	0.938
**SIFT**	**PolyPhen-2_HDIV**	**PolyPhen-2_HVAR**	**MutationTaster2**	**PON-P2**		5491	32.12	808	711	3608	123	0.868	0.835	0.841	0.592	0.532	0.967
**SIFT**	**PolyPhen-2_HDIV**	**PolyPhen-2_HVAR**	**CADD**	**PON-P2**		3759	21.99	944	502	1907	180	0.840	0.792	0.807	0.598	0.653	0.914
**SIFT**	**PolyPhen-2_HDIV**	**MutationTaster2**	**CADD**	**PON-P2**		5815	34.02	994	631	3796	157	0.864	0.857	0.859	0.642	0.612	0.960
**SIFT**	**PolyPhen-2_HVAR**	**MutationTaster2**	**CADD**	**PON-P2**		5661	33.12	1005	608	3656	162	0.861	0.857	0.858	0.646	0.623	0.958
**PolyPhen-2_HDIV**	**PolyPhen-2_HVAR**	**MutationTaster2**	**CADD**	**PON-P2**		5064	29.59	612	558	3525	135	0.819	0.863	0.857	0.576	0.523	0.963
**SIFT**	**PolyPhen-2_HDIV**	**PolyPhen-2_HVAR**	**MutationTaster2**	**CADD**	**PON-P2**	5887	34.44	1027	668	3797	154	0.870	0.850	0.854	0.639	0.606	0.961
