# Supplementary material for: Development of pathogenicity predictors specific for variants that do not comply with clinical guidelines for the use of computational evidence
Source: BMC Genomics. 2017 Aug 11;18(Suppl 5):569. doi: 10.1186/s12864-017-3914-0 (PMC5558188; doi:10.1186/s12864-017-3914-0)
Supplement: Supplementary file 7 — Prediction performance for the PRDIS specific predictors in this work for VS2168 dataset; each corresponds to a different combination of the reference predictors (SIFT, PolyPhen-2, PON-P2, CADD and MutationTaster2). The performance measures are the six standard measures (MCC, accuracy, sensitivity, specificity, PPV and NPV) described in the Materials and Methods section. We also give: the total number and the percentage of cases, and the raw TP, TN, FP and FN values. (PDF 26 kb) [file 12864_2017_3914_MOESM7_ESM.pdf]

PRDIS SPECIFIC PREDICTORS						Number of cases											
						Total	%	TP	FP	TN	FN	SENS	SPEC	ACC	MCC	PPV	NPV
**SIFT**	**PolyPhen-2_HDIV**					41896	13.00	3220	12793	24600	1283	0.715	0.658	0.664	0.238	0.201	0.950
**SIFT**	**PolyPhen-2_HVAR**					35228	10.93	3065	11006	19891	1266	0.708	0.644	0.652	0.236	0.218	0.940
**SIFT**	**MutationTaster2**					86640	26.88	3677	22465	59225	1273	0.743	0.725	0.726	0.237	0.141	0.979
**SIFT**	**CADD**					39485	12.25	3098	6916	27517	1954	0.613	0.799	0.775	0.317	0.309	0.934
**SIFT**	**PON-P2**					15055	4.67	905	1344	11487	1319	0.407	0.895	0.823	0.301	0.402	0.897
**PolyPhen-2_HDIV**	**PolyPhen-2_HVAR**					14733	4.57	800	4531	8889	513	0.609	0.662	0.658	0.161	0.150	0.945
**PolyPhen-2_HDIV**	**MutationTaster2**					77696	24.11	1903	15726	59123	944	0.668	0.790	0.785	0.206	0.108	0.984
**PolyPhen-2_HDIV**	**CADD**					35990	11.17	2424	8892	23679	995	0.709	0.727	0.725	0.275	0.214	0.960
**PolyPhen-2_HDIV**	**PON-P2**					18366	5.70	1132	6850	10024	360	0.759	0.594	0.607	0.194	0.142	0.965
**PolyPhen-2_HVAR**	**MutationTaster2**					81201	25.20	2450	21937	55943	871	0.738	0.718	0.719	0.197	0.100	0.985
**PolyPhen-2_HVAR**	**CADD**					31853	9.88	2173	5789	22642	1249	0.635	0.796	0.779	0.309	0.273	0.948
**PolyPhen-2_HVAR**	**PON-P2**					13529	4.20	1089	3892	8153	395	0.734	0.677	0.683	0.266	0.219	0.954
**MutationTaster2**	**CADD**					72351	22.45	2490	16418	52617	826	0.751	0.762	0.762	0.244	0.132	0.985
**MutationTaster2**	**PON-P2**					41139	12.77	1007	8556	31162	414	0.709	0.785	0.782	0.213	0.105	0.987
**CADD**	**PON-P2**					15252	4.73	631	817	12738	1066	0.372	0.940	0.877	0.334	0.436	0.923
**SIFT**	**PolyPhen-2_HDIV**	**PolyPhen-2_HVAR**				45621	14.16	3632	14407	26171	1411	0.720	0.645	0.653	0.234	0.201	0.949
**SIFT**	**PolyPhen-2_HDIV**	**MutationTaster2**				101399	31.46	4686	25076	70238	1399	0.770	0.737	0.739	0.264	0.157	0.980
**SIFT**	**PolyPhen-2_HDIV**	**CADD**				57872	17.96	4317	11809	39671	2075	0.675	0.771	0.760	0.312	0.268	0.950
**SIFT**	**PolyPhen-2_HDIV**	**PON-P2**				25857	8.02	1179	1962	21129	1587	0.426	0.915	0.863	0.323	0.375	0.930
**SIFT**	**PolyPhen-2_HVAR**	**MutationTaster2**				99738	30.95	4617	22006	71517	1598	0.743	0.765	0.763	0.277	0.173	0.978
**SIFT**	**PolyPhen-2_HVAR**	**CADD**				52545	16.30	3783	8022	38215	2525	0.600	0.827	0.799	0.332	0.320	0.938
**SIFT**	**PolyPhen-2_HVAR**	**PON-P2**				21814	6.77	1471	2364	16710	1269	0.537	0.876	0.833	0.360	0.384	0.929
**SIFT**	**MutationTaster2**	**CADD**				97716	30.32	4992	21893	69259	1572	0.761	0.760	0.760	0.292	0.186	0.978
**SIFT**	**MutationTaster2**	**PON-P2**				48560	15.07	1716	4287	41498	1059	0.618	0.906	0.890	0.370	0.286	0.975
**SIFT**	**CADD**	**PON-P2**				23478	7.29	1503	1888	18589	1498	0.501	0.908	0.856	0.388	0.443	0.925
**PolyPhen-2_HDIV**	**PolyPhen-2_HVAR**	**MutationTaster2**				86807	26.94	2585	19395	63672	1155	0.691	0.767	0.763	0.214	0.118	0.982
**PolyPhen-2_HDIV**	**PolyPhen-2_HVAR**	**CADD**				41288	12.81	2653	8772	28439	1424	0.651	0.764	0.753	0.277	0.232	0.952
**PolyPhen-2_HDIV**	**PolyPhen-2_HVAR**	**PON-P2**				19345	6.00	1374	7413	10201	357	0.794	0.579	0.598	0.214	0.156	0.966
**PolyPhen-2_HDIV**	**MutationTaster2**	**CADD**				92853	28.81	3485	18166	69906	1296	0.729	0.794	0.790	0.273	0.161	0.982
**PolyPhen-2_HDIV**	**MutationTaster2**	**PON-P2**				49033	15.21	1116	4672	42418	827	0.574	0.901	0.888	0.287	0.193	0.981
**PolyPhen-2_HDIV**	**CADD**	**PON-P2**				24844	7.71	1609	4727	17853	655	0.711	0.791	0.783	0.331	0.254	0.965
**PolyPhen-2_HVAR**	**MutationTaster2**	**CADD**				92539	28.71	4129	28034	59485	891	0.823	0.680	0.687	0.239	0.128	0.985
**PolyPhen-2_HVAR**	**MutationTaster2**	**PON-P2**				46907	14.56	1084	4353	40530	940	0.536	0.903	0.887	0.278	0.199	0.977
**PolyPhen-2_HVAR**	**CADD**	**PON-P2**				21080	6.54	1361	2515	16313	891	0.604	0.866	0.838	0.375	0.351	0.948
**MutationTaster2**	**CADD**	**PON-P2**				46398	14.40	1411	5442	38755	790	0.641	0.877	0.866	0.310	0.206	0.980
**SIFT**	**PolyPhen-2_HDIV**	**PolyPhen-2_HVAR**	**MutationTaster2**			103895	32.24	4917	24508	72892	1578	0.757	0.748	0.749	0.272	0.167	0.979
**SIFT**	**PolyPhen-2_HDIV**	**PolyPhen-2_HVAR**	**CADD**			59850	18.57	4145	9754	43358	2593	0.615	0.816	0.794	0.323	0.298	0.944
**SIFT**	**PolyPhen-2_HDIV**	**PolyPhen-2_HVAR**	**PON-P2**			26280	8.15	1369	2446	20937	1528	0.473	0.895	0.849	0.327	0.359	0.932
**SIFT**	**PolyPhen-2_HDIV**	**MutationTaster2**	**CADD**			106920	33.18	5373	20276	79356	1915	0.737	0.796	0.792	0.315	0.209	0.976
**SIFT**	**PolyPhen-2_HDIV**	**MutationTaster2**	**PON-P2**			53117	16.48	1875	4261	45807	1174	0.615	0.915	0.898	0.386	0.306	0.975
**SIFT**	**PolyPhen-2_HDIV**	**CADD**	**PON-P2**			30367	9.42	1997	3183	23878	1309	0.604	0.882	0.852	0.403	0.386	0.948
**SIFT**	**PolyPhen-2_HVAR**	**MutationTaster2**	**CADD**			104913	32.55	4797	13392	84158	2566	0.652	0.863	0.848	0.347	0.264	0.970
**SIFT**	**PolyPhen-2_HVAR**	**MutationTaster2**	**PON-P2**			51153	15.87	1899	3633	44438	1183	0.616	0.924	0.906	0.414	0.343	0.974
**SIFT**	**PolyPhen-2_HVAR**	**CADD**	**PON-P2**			27112	8.41	2160	3327	20505	1120	0.659	0.860	0.836	0.421	0.394	0.948
**SIFT**	**MutationTaster2**	**CADD**	**PON-P2**			51252	15.90	1635	2071	45883	1663	0.496	0.957	0.927	0.429	0.441	0.965
**PolyPhen-2_HDIV**	**PolyPhen-2_HVAR**	**MutationTaster2**	**CADD**			97258	30.18	3585	15087	76808	1778	0.668	0.836	0.827	0.292	0.192	0.977
**PolyPhen-2_HDIV**	**PolyPhen-2_HVAR**	**MutationTaster2**	**PON-P2**			49854	15.47	1701	11838	35860	455	0.789	0.752	0.753	0.247	0.126	0.987
**PolyPhen-2_HDIV**	**PolyPhen-2_HVAR**	**CADD**	**PON-P2**			25478	7.91	1683	4331	18712	752	0.691	0.812	0.800	0.348	0.280	0.961
**PolyPhen-2_HDIV**	**MutationTaster2**	**CADD**	**PON-P2**			51788	16.07	2056	10676	38556	500	0.804	0.783	0.784	0.296	0.161	0.987
**PolyPhen-2_HVAR**	**MutationTaster2**	**CADD**	**PON-P2**			49742	15.43	1871	5700	41444	727	0.720	0.879	0.871	0.371	0.247	0.983
**SIFT**	**PolyPhen-2_HDIV**	**PolyPhen-2_HVAR**	**MutationTaster2**	**CADD**		108554	33.68	5265	16554	84398	2337	0.693	0.836	0.826	0.337	0.241	0.973
**SIFT**	**PolyPhen-2_HDIV**	**PolyPhen-2_HVAR**	**MutationTaster2**	**PON-P2**		53471	16.59	2097	4774	45524	1076	0.661	0.905	0.891	0.400	0.305	0.977
**SIFT**	**PolyPhen-2_HDIV**	**PolyPhen-2_HVAR**	**CADD**	**PON-P2**		30688	9.52	1879	2790	24487	1532	0.551	0.898	0.859	0.393	0.402	0.941
**SIFT**	**PolyPhen-2_HDIV**	**MutationTaster2**	**CADD**	**PON-P2**		54912	17.04	2343	4719	46687	1163	0.668	0.908	0.893	0.421	0.332	0.976
**SIFT**	**PolyPhen-2_HVAR**	**MutationTaster2**	**CADD**	**PON-P2**		53085	16.47	2600	5453	44108	924	0.738	0.890	0.880	0.436	0.323	0.979
**PolyPhen-2_HDIV**	**PolyPhen-2_HVAR**	**MutationTaster2**	**CADD**	**PON-P2**		52358	16.25	2013	7387	42253	705	0.741	0.851	0.845	0.342	0.214	0.984
**SIFT**	**PolyPhen-2_HDIV**	**PolyPhen-2_HVAR**	**MutationTaster2**	**CADD**	**PON-P2**	55196	17.13	2526	4962	46625	1083	0.700	0.904	0.890	0.436	0.337	0.977
