# Supplementary material for: Development of pathogenicity predictors specific for variants that do not comply with clinical guidelines for the use of computational evidence
Source: BMC Genomics. 2017 Aug 11;18(Suppl 5):569. doi: 10.1186/s12864-017-3914-0 (PMC5558188; doi:10.1186/s12864-017-3914-0)
Supplement: Supplementary file 17 — Prediction performance for the hybrid predictor. We give the raw TP, TN, FP and FN values and the values of the six standard measures (MCC, accuracy, sensitivity, specificity, PPV and NPV) described in the Materials and Methods section. (PDF 24 kb) [file 12864_2017_3914_MOESM17_ESM.pdf]

Hybrid Models						TP	FP	TN	FN	SENS	SPEC	ACC	MCC	PPV	NPV
SIFT	PolyPhen-2_HDIV					13542	8237	33914	1656	0.891	0.805	0.827	0.633	0.622	0.953
SIFT	PolyPhen-2_HVAR					13213	6906	35255	1980	0.870	0.836	0.845	0.653	0.657	0.947
SIFT	MutationTaster2					8762	5551	17615	813	0.915	0.760	0.806	0.619	0.612	0.956
SIFT	CADD					8254	3211	19955	1321	0.862	0.861	0.862	0.690	0.720	0.938
SIFT	PON-P2					8730	2937	17241	578	0.938	0.854	0.881	0.753	0.748	0.968
PolyPhen-2_HDIV	PolyPhen-2_HVAR					13940	9749	33772	1594	0.897	0.776	0.808	0.605	0.588	0.955
PolyPhen-2_HDIV	MutationTaster2					9035	6285	16893	540	0.944	0.729	0.792	0.613	0.590	0.969
PolyPhen-2_HDIV	CADD					8624	3830	19348	951	0.901	0.835	0.854	0.689	0.692	0.953
PolyPhen-2_HDIV	PON-P2					9020	3816	17184	343	0.963	0.818	0.863	0.731	0.703	0.980
PolyPhen-2_HVAR	MutationTaster2					8881	5179	17991	682	0.929	0.776	0.821	0.648	0.632	0.963
PolyPhen-2_HVAR	CADD					8521	3515	19667	1050	0.890	0.848	0.861	0.697	0.708	0.949
PolyPhen-2_HVAR	PON-P2					8917	3284	17740	446	0.952	0.844	0.877	0.750	0.731	0.975
MutationTaster2	CADD					8789	5727	17460	789	0.918	0.753	0.801	0.614	0.605	0.957
MutationTaster2	PON-P2					5609	1810	9368	177	0.969	0.838	0.883	0.772	0.756	0.981
CADD	PON-P2					5510	1246	9927	290	0.950	0.888	0.910	0.812	0.816	0.972
SIFT	PolyPhen-2_HDIV	PolyPhen-2_HVAR				13431	7675	34472	1757	0.884	0.818	0.835	0.642	0.636	0.952
SIFT	PolyPhen-2_HDIV	MutationTaster2				8880	4821	18336	692	0.928	0.792	0.832	0.663	0.648	0.964
SIFT	PolyPhen-2_HDIV	CADD				8444	3116	20041	1128	0.882	0.865	0.870	0.712	0.730	0.947
SIFT	PolyPhen-2_HDIV	PON-P2				8689	2956	17174	483	0.947	0.853	0.883	0.759	0.746	0.973
SIFT	PolyPhen-2_HVAR	MutationTaster2				8807	4519	18639	756	0.921	0.805	0.839	0.672	0.661	0.961
SIFT	PolyPhen-2_HVAR	CADD				8387	2924	20240	1180	0.877	0.874	0.875	0.718	0.741	0.945
SIFT	PolyPhen-2_HVAR	PON-P2				8636	2731	17414	533	0.942	0.864	0.889	0.767	0.760	0.970
SIFT	MutationTaster2	CADD				8725	4710	18456	850	0.911	0.797	0.830	0.655	0.649	0.956
SIFT	MutationTaster2	PON-P2				5563	1481	9635	209	0.964	0.867	0.900	0.799	0.790	0.979
SIFT	CADD	PON-P2				5450	1075	10055	321	0.944	0.903	0.917	0.826	0.835	0.969
PolyPhen-2_HDIV	PolyPhen-2_HVAR	MutationTaster2				8975	5757	17407	587	0.939	0.751	0.806	0.631	0.609	0.967
PolyPhen-2_HDIV	PolyPhen-2_HVAR	CADD				8534	3509	19661	1032	0.892	0.849	0.861	0.699	0.709	0.950
PolyPhen-2_HDIV	PolyPhen-2_HVAR	PON-P2				8964	3534	17457	393	0.958	0.832	0.871	0.741	0.717	0.978
PolyPhen-2_HDIV	MutationTaster2	CADD				8872	4802	18376	703	0.927	0.793	0.832	0.664	0.649	0.963
PolyPhen-2_HDIV	MutationTaster2	PON-P2				5599	1603	9522	174	0.970	0.856	0.895	0.792	0.777	0.982
PolyPhen-2_HDIV	CADD	PON-P2				5523	1192	9945	247	0.957	0.893	0.915	0.824	0.822	0.976
PolyPhen-2_HVAR	MutationTaster2	CADD				8783	4348	18828	784	0.918	0.812	0.843	0.678	0.669	0.960
PolyPhen-2_HVAR	MutationTaster2	PON-P2				5568	1391	9745	200	0.965	0.875	0.906	0.810	0.800	0.980
PolyPhen-2_HVAR	CADD	PON-P2				5477	1074	10076	289	0.950	0.904	0.919	0.831	0.836	0.972
MutationTaster2	CADD	PON-P2				5586	1544	9607	191	0.967	0.862	0.898	0.796	0.783	0.981
SIFT	PolyPhen-2_HDIV	PolyPhen-2_HVAR	MutationTaster2			8788	4385	18768	774	0.919	0.811	0.842	0.677	0.667	0.960
SIFT	PolyPhen-2_HDIV	PolyPhen-2_HVAR	CADD			8483	3167	19987	1081	0.887	0.863	0.870	0.713	0.728	0.949
SIFT	PolyPhen-2_HDIV	PolyPhen-2_HVAR	PON-P2			8550	2512	17616	617	0.933	0.875	0.893	0.773	0.773	0.966
SIFT	PolyPhen-2_HDIV	MutationTaster2	CADD			8696	3858	19299	876	0.908	0.833	0.855	0.694	0.693	0.957
SIFT	PolyPhen-2_HDIV	MutationTaster2	PON-P2			5560	1319	9776	203	0.965	0.881	0.910	0.816	0.808	0.980
SIFT	PolyPhen-2_HDIV	CADD	PON-P2			5466	1069	10039	297	0.948	0.904	0.919	0.830	0.836	0.971
SIFT	PolyPhen-2_HVAR	MutationTaster2	CADD			8723	3999	19161	842	0.912	0.827	0.852	0.690	0.686	0.958
SIFT	PolyPhen-2_HVAR	MutationTaster2	PON-P2			5517	1179	9927	242	0.958	0.894	0.916	0.826	0.824	0.976
SIFT	PolyPhen-2_HVAR	CADD	PON-P2			5479	1067	10052	281	0.951	0.904	0.920	0.832	0.837	0.973
SIFT	MutationTaster2	CADD	PON-P2			5529	1326	9787	240	0.958	0.881	0.907	0.810	0.807	0.976
PolyPhen-2_HDIV	PolyPhen-2_HVAR	MutationTaster2	CADD			8873	4852	18319	693	0.928	0.791	0.831	0.662	0.646	0.964
PolyPhen-2_HDIV	PolyPhen-2_HVAR	MutationTaster2	PON-P2			5582	1488	9631	185	0.968	0.866	0.901	0.802	0.790	0.981
PolyPhen-2_HDIV	PolyPhen-2_HVAR	CADD	PON-P2			5510	1161	9972	255	0.956	0.896	0.916	0.826	0.826	0.975
PolyPhen-2_HDIV	MutationTaster2	CADD	PON-P2			5579	1442	9679	191	0.967	0.870	0.903	0.806	0.795	0.981
PolyPhen-2_HVAR	MutationTaster2	CADD	PON-P2			5547	1294	9839	220	0.962	0.884	0.910	0.817	0.811	0.978
SIFT	PolyPhen-2_HDIV	PolyPhen-2_HVAR	MutationTaster2	CADD		8673	3804	19351	891	0.907	0.836	0.857	0.695	0.695	0.956
SIFT	PolyPhen-2_HDIV	PolyPhen-2_HVAR	MutationTaster2	PON-P2		5559	1353	9741	199	0.965	0.878	0.908	0.813	0.804	0.980
SIFT	PolyPhen-2_HDIV	PolyPhen-2_HVAR	CADD	PON-P2		5473	1078	10030	286	0.950	0.903	0.919	0.830	0.835	0.972
SIFT	PolyPhen-2_HDIV	MutationTaster2	CADD	PON-P2		5534	1235	9858	229	0.960	0.889	0.913	0.821	0.818	0.977
SIFT	PolyPhen-2_HVAR	MutationTaster2	CADD	PON-P2		5518	1182	9921	242	0.958	0.894	0.916	0.825	0.824	0.976
PolyPhen-2_HDIV	PolyPhen-2_HVAR	MutationTaster2	CADD	PON-P2		5550	1313	9804	215	0.963	0.882	0.909	0.815	0.809	0.979
SIFT	PolyPhen-2_HDIV	PolyPhen-2_HVAR	MutationTaster2	CADD	PON-P2	5533	1238	9855	226	0.961	0.888	0.913	0.821	0.817	0.978
